# Crossability of *Triticum urartu* and *Triticum monococcum* Wheats, Homoeologous Recombination, and Description of a Panel of Interspecific Introgression Lines

**DOI:** 10.1534/g3.114.013623

**Published:** 2014-08-21

**Authors:** Agostino Fricano, Andrea Brandolini, Laura Rossini, Pierre Sourdille, Joerg Wunder, Sigi Effgen, Alyssa Hidalgo, Daniela Erba, Pietro Piffanelli, Francesco Salamini

**Affiliations:** * Parco Tecnologico Padano, 26900 Lodi, Italy; † Department of Agricultural and Environmental Sciences (DiSAA), Università degli Studi di Milano, 20133 Milan, Italy; ‡ Consiglio per la Ricerca e la Sperimentazione in Agricoltura - Unità di Ricerca per la Selezione dei Cereali e la Valorizzazione delle varietà vegetali (CRA-SCV), 26866 S. Angelo Lodigiano (LO), Italy; § Institute National de la Recherche Agronomique, UMR1035 Clermont-Ferrand, France; ** Research and Innovation Centre, Fondazione Edmund Mach, 38010 San Michele all’Adige, Trento, Italy; †† Max-Planck-Institut für Pflanzenzuchtungforschung, 50829 Cologne, Germany; ‡‡ Department of Food, Environmental and Nutritional Sciences (DeFENS), Università degli Studi di Milano, 20133 Milan, Italy

**Keywords:** chromosomes recombination, diploid wheats, fertility, interspecific introgression lines

## Abstract

*Triticum monococcum* (genome A^m^) and *T. urartu* (genome A^u^) are diploid wheats, with the first having been domesticated in the Neolithic Era and the second being a wild species. In a germplasm collection, rare wild *T. urartu* lines with the presence of *T. monococcum* alleles were found. This stimulated our interest to develop interspecific introgression lines of *T. urartu* in *T. monococcum*, a breeding tool currently implemented in several crop species. Moreover, the experiments reported were designed to reveal the existence in nature of A^m^/A^u^ intermediate forms and to clarify whether the two species are at least marginally sexually compatible. From hand-made interspecific crosses, almost-sterile F_1_ plants were obtained when the seed-bearing parent was *T. monococcum*. A high degree of fertility was, however, evident in some advanced generations, particularly when *T. urartu* donors were molecularly more related to *T. monococcum*. Analysis of the marker populations demonstrated chromosome pairing and recombination in F_1_ hybrid plants. Forty-six introgression lines were developed using a line of *T. monococcum* with several positive agronomic traits as a recurrent parent. Microsatellite markers were tested on A^u^ and A^m^ genomes, ordered in a *T. monococcum* molecular map, and used to characterize the exotic DNA fragments present in each introgression line. In a test based on 28 interspecific introgression lines, the existence of genetic variation associated with *T. urartu* chromosome fragments was proven for the seed content of carotenoids, lutein, β-cryptoxanthin, and zinc. The molecular state of available introgression lines is summarized.

*Triticum urartu* Thum. ex Gandil., the genome A donor of durum and bread wheats, and *Triticum monococcum* L. have the same chromosome number and similar genome size and gene content (summarized in Özkan *et al.* 2010). The two species have a long history of coexistence in a primary distribution area (Zohary and Hopf 2000). Within *T. monococcum*, two subspecies are recognized: the wild *T. monococcum* ssp. *boeoticum* Boiss. (*T. m. boeoticum*) and its domesticated form *T. monococcum* ssp. *monococcum* (*T. m. monococcum*); intermediate feral genotypes derived from hybrids between wild and domesticated forms are included under the taxon *T. monococcum* ssp. *aegilopoides (T. m. aegilopoides)* (Salamini *et al.* 2002).

A^m^ and A^u^ genomes have a high level of gene colinearity (Devos *et al.* 1995), but molecular differences have been found (Wicker *et al.* 2003). Some of these differences should be responsible for the almost absolute sterility of the hybrids between *T. urartu* and *T. m. boeoticum*, a condition that suggested establishing them as distinct species (Johnson and Dhaliwal 1976). Differences in hybrid seed setting and viability in reciprocal crosses of the two species have been attributed to the cytoplasm (Johnson and Dhaliwal 1976) or to endosperm development (Dhaliwal 1977a), including a pollen factor controlling endosperm abortion (Gill and Waines 1978).

Macrocolinearity among related taxa is inferred when a similar marker order is observed in mapping experiments with 10- to 20-cM resolution (Lu and Faris 2006). Intrachromosomal rearrangements and translocations may, however, break macrocolinearity. In the *Gramineae*, macrocolinearity has a significant level of conservation among species of subfamilies *Panicoideae*, *Triticeae*, *Pooideae*, and *Ehrhartoideae* (Moore *et al.* 1995; Keller and Feuillet 2000; Bolot *et al.* 2009; Salse and Feuillet 2011).

Introgression lines are largely isogenic genotypes, but each one contains a well-defined homozygous chromosome segment of a donor parent (Eshed and Zamir 1994). When the donor parent is a different species, they are named interspecific introgression lines. Introgression lines are developed by backcrossing hybrid progenies to the recurrent parent. From their introduction as a tool for QTL and gene mapping (Eshed and Zamir 1994), introgression lines have been largely used in the analysis of related genomes (Holtan and Hake 2003; Baxter *et al.* 2005; Lippman *et al.* 2007; Schmalenbach *et al.* 2008; Timonova *et al.* 2013). They have been developed in maize (Szalma *et al.* 2007), rice (Fukuta *et al.* 2012), rye (Falke *et al.* 2008, 2009; Mahone *et al.* 2013), wheat (Timonova *et al.* 2013), and, particularly, in barley to investigate agronomic traits, heading time and response to biotic stresses (Matus *et al.* 2003; Schmalenbach *et al.* 2008, 2009).

The objectives of this work were to: i) deepen the knowledge of the molecular diversity of *T. monococcum* and *T. urartu*; ii) clarify whether the two species are at least marginally sexually compatible; iii) investigate genome-wide macrocolinearity relationships between the A^m^ and A^u^ genomes; and iv) develop a panel of interspecific introgression lines of *T. urartu* in *T. monococcum*, a tool of relevant value when characterizing the contribution of *T. urartu* to the durum and bread wheat genomes

## Materials and Methods

### Diploid wheat accessions evaluated and assessment of their variation

Four hundred ninety-six diploid wheat accessions were considered. The 330 *T. monococcum* accessions (66 ssp. *monococcum*, nine ssp. *aegilopoides*, and 255 ssp. *boeoticum*) are described in Heun *et al.* (1997). The 166 *T. urartu* samples were provided by the Institut für Genetik und Kulturpflanzen Forschung, Gatersleben, Germany; the Cambridge Laboratory, Norwich, UK; the Vavilov All Union Institute of Plant Industry, Saint Petersburg, Russia; the Kansas State University, Kansas, USA; and the National Small Grains Collection, Idaho, USA. The locations of sampling of all the diploid wheats considered is summarized in part in Supporting Information, Figure S1, and a list of the 496 accessions with their origin is detailed in Table S4. The accessions considered cover the primary and secondary distribution ranges of the two A genome species (Figure S1). Most wild *T. m. boeoticum* samples were from primary habitats in the central-eastern parts of the Fertile Crescent. Sixty eight samples of domesticated einkorn (*T*. *m. monococcum*), mainly from Europe, and nine *T*. *m. aegilopoides* from the Balkans were included. For *T. urartu*, 91 accessions were collected from the western part and 74 from the central-eastern part of the Fertile Crescent; the five accessions from Armenia may represent cases of colonization of segetal habitats. Passport data of *T. urartu* accessions (Valkoun *et al.* 1998) support the sampling in primary habitats.

### DNA extraction and amplified fragment-length polymorphism (AFLP) analysis

DNA extraction from 7-d-old seedlings followed a modified CTAB procedure (Murray and Thompson 1980). AFLP analysis was as in Vos *et al.* (1995), using the AFLP combinations E36-M36, E37-M40, E42M32, E42-M33, E42-M38, E40-M40, and E40-M38. For each wheat line, the presence and absence of amplified fragments was scored, considering only unambiguous electrophoretic readings. From the AFLP matrix, pair-wise genetic distances among accessions were computed as in Jaccard (1908). Principal coordinates analysis was performed on genetic distance data. Phylogenetic analyses were based on the computer package NTSYS pc V. 2.1 (Rohlf 2000).

### SSR linkage mapping

A linkage map based on SSR markers was developed starting with 121 F_2_ plants from the cross between ID69, a free-threshing cultivated einkorn (*T. m*. ssp. *monococcum* var. *sinskajae*), and ID49, a wild einkorn line *(T. m*. ssp. *boeoticum*). An independent F_2_ population derived from the same cross was considered in the study of Taenzler *et al.* (2002). The allelic state of codominant microsatellite markers was assessed for *T. m*. ssp. *boeoticum* ID49, *T. monococcum* ssp. *monococcum* var. *sinskajae* ID69, but also for the accessions *T. monococcum* ssp. *monococcum* L118, and *T. urartu* ID388, later on used to develop the interspecific introgression lines.

Three hundred sixty mapped SSR markers were considered, including GWM markers (Röder *et al.* 1998), GDM markers (Pestsova *et al.* 2000), WMC markers (Gupta *et al.* 2002), CFD markers (Guyomarc’h *et al.* 2002), CFA markers (Sourdille *et al.* 2003), and BARC markers (Song *et al.* 2005). In addition, 180 SSRs from the GPW set (Sourdille *et al.* 2010) also were introduced. As the last SSRs were not previously published, their DNA primers are reported in the Table S2. Labeling of polymerase chain reaction (PCR) fragments with ABI dyes followed the three-primer system (Schuelke 2000). Amplification products for different SSR markers, labeled with ABI dyes 6-FAM, NED, PET, and VIC, were pooled and separated by capillary electrophoresis on a DNA Analyzer ABI3730 (Applied Biosystems, Foster City, CA). Fragment analysis was carried out in GeneMapper 4.0 (Applied Biosystems). A panel of 170 of 317 informative SSR markers was used to genotype the DNA samples of the mapping population. Linkage analysis as well as statistical tests to assess segregation ratios were performed with JoinMap 4.0 (Kyazma, 6700 AD Wageningen, the Netherlands), using a recombination frequency from 0.250 to 0.050 to create groups, and a logarithm of the odds (LOD) score >3 to map microsatellite loci. Kosambi’s mapping function was used to calculate genetic distances.

### *T. monococcum* x *T. urartu* crosses and interspecific recombination between *T. monococcum* and *T. urartu* chromosomes

Crosses were carried out with *T. monococcum* or *T. boeoticum* as one parent and *T. urartu* as the other, including reciprocals to evaluate maternal and paternal species-specific effects on fertility (Table 2). The rationale behind the choice of parents was to control whether genetic relatedness among accessions of the two *taxa* has the potential to improve hybrid fertility. Fertility was evaluated as the ratio between the observed number of seeds and the number of florets per spikelet.

Recombination events between *T. monococcum* and *T. urartu* chromosomes were analyzed in two different types of recombinant families. First, two F_2_ selfed seeds recovered from more than 3000 spikelets of the hybrid between the female parent ID396 (*T. m*. ssp. *monococcum)* and ID1122 (the *T. urartu* line showing some *T. monococcum* introgression) gave rise to two F_2_ plants from which the F_3_ segregating populations B53 (280 S_2_ plants) and B54 (80 S_2_ plants) were derived. The role of foreign pollen in the genetic origin of the two F_2_ seeds was excluded by AFLP analysis. Second, the advanced *T. m*. ssp. *monococcum* line L118, an improved free-threshing, short straw einkorn line developed for breeding purposes, was mated as female parent to the *T. urartu* accession ID388. The F_1_ plants were self-fertilized and the five derived F_2_ plants backcrossed to L118 to obtain 71 lines. In addition, 48 lines were derived from backcrosses to the F_1_ without a step of self-fertilization. The lines were subsequently backcrossed four times to L118. The offspring were fingerprinted using a panel of 155 microsatellites mapped in the ID69 × ID49 segregating population. Plant lines bearing nonredundant chromosome segments of *T. urartu* were selected. The panel of codominant markers used in fingerprinting was based on microsatellite markers mapping to the A genome of hexaploid wheat. The markers were tested on *T. m* ssp. *monococcum* L118 and ID69, *T. urartu* ID388, and *T. monococcum* ssp. *boeoticum* ID49 and subsequently mapped on the ID49 × ID69 segregating population (Taenzler *et al.* 2002). Among more than 300 informative SSRs, 170 were selected to fingerprint the 121 F_2_ plants.

For the B53 and B54 F_3_ segregating lines, AFLP markers were used to assess genetic recombination on 140 B53 lines and on all 80 B54 lines. For B53, we used the 27 primer combinations E32M60, E32M61, E35M59, E35M60, E35M61, *E36M36*, E36M38, *E36M40*, E36M62, E37M32, *E37M33*, *E37M38*, *E37M40*, E37M48, E37M60, E37M61, E38M61, E40M32, *E40M38*, *E40M40*, E41M32, *E41M33*, *E41M38*, E41M40, E42M32, *E42M38*, and *E42M40*; the 11 combinations in italics were used in the case of the B54 population. In the case of a molecular marker map deriving from an intraspecific hybrid population, chromosomal regions with distorted segregation ratios are expected (Jenczewski *et al.* 1997; Yin *et al.* 2004) but despite this, maps can still be created (Gebhardt *et al.* 2005). In our case, the existence and level of DNA interchange among A^m^ and A^u^ chromosomes was implemented based on the following steps.

Step 1. In the B53 and B54 AFLP databases, amplified fragments were assigned specifically to *T. monococcum* when absent in *T. urartu*, and the reverse.
Step 2. The database was queried for AFLP fragments assigned to linkage groups in the *T. monococcum* map of Taenzler *et al.* (2002). Groups of 1 to 12 A^m^ or A^u^ fragments present in populations B53 and B54 and mapping to the same linkage group interval were interpreted as chromosomal regions homozygous in B53 and B54, derived either from *T. monococcum* or from *T. urartu*. AFLP fragments present in *T. urartu*, identical in size with A^m^ fragments segregating in the *T. monococcum* crosses of Taenzler *et al.* (2002), were considered homeologous, and thus anchored in the Taenzler *et al.* (2002) backbone map to the corresponding linkage regions.
Step 3. Fragments segregating in B53 and B54 were analyzed by the software MAPMAKER/EXP Version 3.0 (Lincoln *et al.* 1993). A large number of independent groups of fragments linked through null or reduced recombination values was observed (linkage subgroups). Subgroups were chosen with at least a polymorphism mapping to a specific linkage region of the map of Taenzler *et al.* (2002). Within subgroups including both A^m^ and A^u^ fragments, two cases were distinguished.

Repulsion between linked A^m^ and A^u^ fragments: in this case two clusters of A^m^ and A^u^ fragments were anchored to the same syntenic region of the backbone map. The region was, accordingly, considered heterozygous for *T. monococcum* and *T. urartu* DNA.

Coupling between A^m^ and A^u^ fragments: this finding was interpreted as if in one of the two gametes that generated the two original hybrid seeds, a crossing-over event in the F_1_ plants created a *T. monococcum-T. urartu* mosaic chromosome, bordered by A^m^ and A^u^ polymorphisms. Orange bars in Figure 2 mark these events, as well as segments hosting those groups of bands described in Figure S2.

Step 4. The anchoring to the *T. monococcum* backbone map of the regions hosting both homozygous A^m^ or A^u^ or heterozygous A^m^-A^u^ clusters of fragments was based on AFLP polymorphisms found both in the populations B53 and B 54 and in the map of Taenzler *et al.* (2002). After anchoring, the resulting map consisted of chromosomal segments within which recombination took place. These were probably separated by recombination gaps of unknown size.

In the second approach, evidence was provided for the existence of recombination between A^u^ and A^m^ chromosomes, together with the development of interspecific introgression lines. The offspring of the cross L118 × ID388 were fingerprinted, as described, during each successive cycle of back-crossing, and lines bearing nonredundant chromosome segments of *T. urartu* were selected.

### Macrocolinearity of A^u^ and A^m^ genomes

A macrocolinearity analysis was carried out to reveal occurrence of major chromosome rearrangements during or after the separation of *T. urartu* from *T. monococcum* (starting from 0.5 to 2 million years ago; Wicker *et al.* 2003). The loci mapped in the ID69 × ID49 segregating population were compared, in terms of map positions, with the corresponding loci mapped on the A genome of the hexaploid wheat, using Circos software (Krzywinski *et al.* 2009). In this analysis (Figure 4), map positions of microsatellite loci in the two species are joined by bridges which, according to the colors adopted, may indicate homeologous or nonhomeologous relationships.

### Quantification of carotenoids, tocols, and mineral micronutrients

Only field testing of interspecific introgression lines would assess the agronomic and breeding values of the introgression lines. However, because such experiments are a long way from being carried out, we have introduced a preliminary analysis to at least quantify the existence of phenotypic variation associated with the *T. urartu* chromosome fragments present in the introgression lines. Antioxidants and micronutrient contents of introgression lines seeds were chosen as test characters. Twenty-eight introgression lines were considered and evaluated for their kernel levels of the antioxidants lutein, α- + β-carotene, β-cryptoxanthin, zeaxanthin, α-tocopherol, β-tocopherol, α-tocotrienol, β-tocotrienol, and of the minerals zinc, iron, and calcium (Table 3). Two replications of 20 g of seeds of each line grown under standard agronomic conditions (Castagna *et al.* 1995) were considered. Carotenoids quantification was by normal-phase high-performance liquid chromatography as described by Hidalgo *et al.* (2010), whereas tocopherols and tocotrienols quantification was by normal-phase high-performance liquid chromatography as described by Hidalgo and Brandolini (2010). Measurements were duplicated, starting from the two samples of whole meal; the results are presented as means, expressed as mg/kg dry matter (DM).

Mineral concentrations of Zn, Fe, and Ca were determined by Atomic Absorption (AAnalyst 800; PerkinElmer, Waltham, MA) following Erba *et al.* (2011). All the analyses were carried out on two whole meal samples per introgression line; the results were expressed as means on a dry matter basis (mg/kg DM).

Variation in carotenoids, tocols, and microelements in parents and derived introgression lines was analyzed by one-way analysis of variance and the general standard error was used in the *t*-test to calculate the significance of differences among lines and parents.

## Results

### Molecular variation in the gene pool of A genome wheats

**Figure 1:**
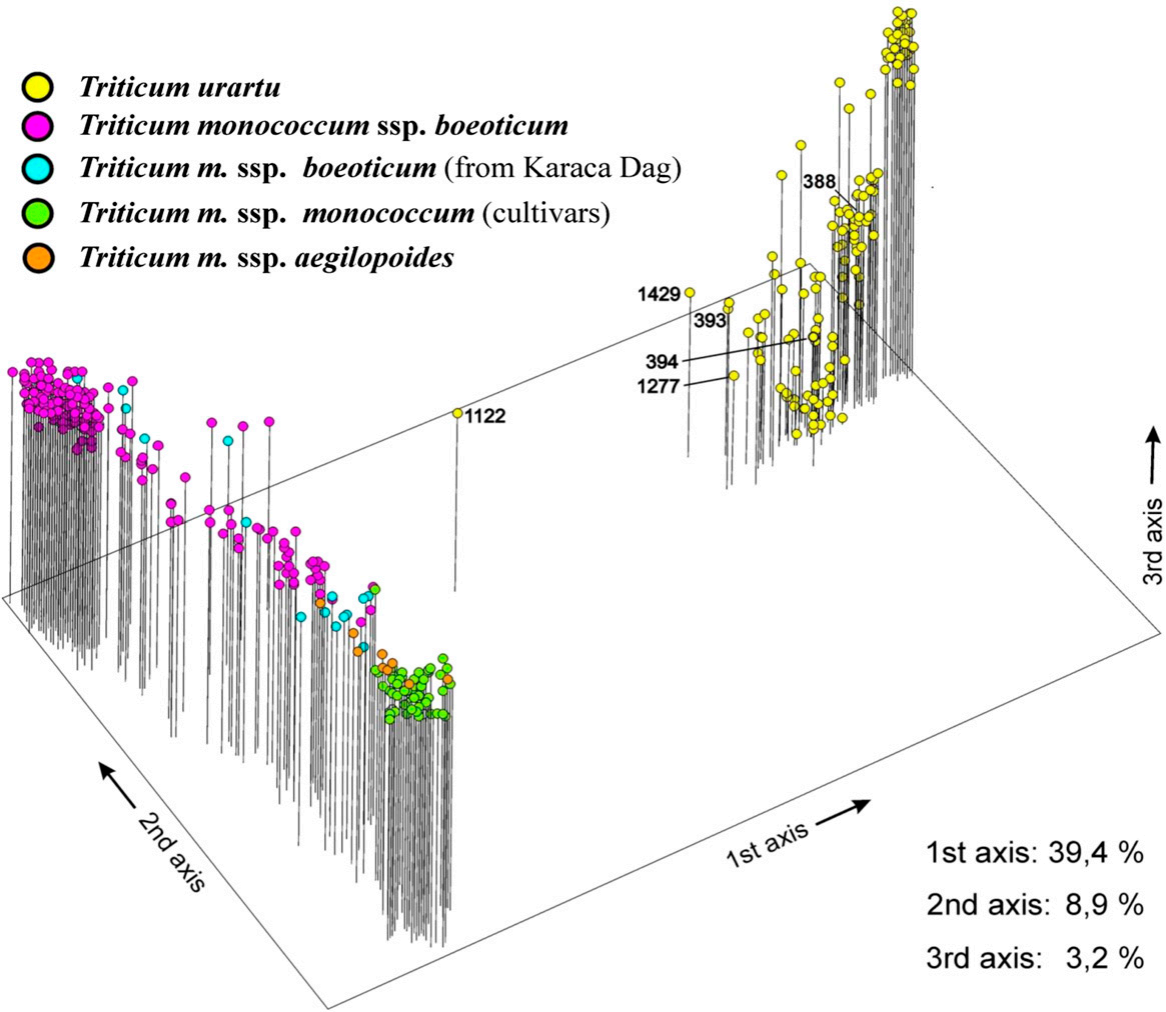
Principal coordinates analysis of the diploid wheat accessions considered in this study, based on 257 amplified fragment-length polymorphism markers. Two main groups or clades encompassing *T. urartu* (yellow) and *T. monococcum* ssp. *monococcum* (green) are evident. Within *T. monococcum*, ssp. *boeoticum* (purple), ssp. *boeoticum* from the KarakaDag area (blue), and ssp. *aegilopoides* (orange) accessions are indicated.

A total of 257 AFLP fragments revealed polymorphisms in at least one accession of the two species (Table 1A). Thirty-five were present only in the einkorn gene pool (wild and domesticated), whereas 25 were exclusive to *T. urartu*. The principal coordinates analysis plot (Figure 1) displays two main, well-supported clusters, each corresponding to a species. The first three principal coordinates explained 39.4%, 8.9%, and 3.2% of the AFLP variation, respectively. The cultivated einkorns and their feral forms cluster at one end of the field of variation, whereas the wild *T. m. boeoticum* lines group preferentially at the other end. In *T. monococcum* a continuum leading gradually from the wild to the domesticated forms is evident: the wild samples closest to the domestic einkorn were sampled in the KarakaDag mountain range. The results indicate that few *T. urartu* accessions, like ID 1122, ID 1429, ID393, and ID 1277, have an intermediate topology between *T. urartu* and *T. monococcum*. They may represent cases of gene flow between the two *taxa*. The AFLP profiles of these accessions (Table 1B) show that they are characterized, in different measure, by the simultaneous presence of those AFLP fragments defined as einkorn- and *T. urartu*-specific.

**Table 1:** AFLP polymorphic loci specific for *T. monococcum* or *T. urartu*

A. Average number of *T. monococcum* or *T. urartu* specific polymorphic loci		
1	2	3
Total number of polymorphic loci either in M or U or both	No. of loci polymorphic in 338 *T. monococcum**^a^* lines with homozygous null alleles *T. urartu* lines*^b^*	No. of loci polymorphic in 168 *T. urartu**^b^* lines with homozygous null alleles in 338 T. *monococcum* lines*^a^*
257	35	25

B. Average number of *T. monococcum* (M) or *T. urartu* (U) specific loci, as defined in Table 1A, columns 2 (M) and 3 (U) (T = total loci), present in																				
Standard *T. monococcum* lines*^c^*			Intermediate Lines*^d^*															Standard *T. urartu* Lines*^e^*		
			ID1122			ID1429			ID393			ID1277			ID394					
M	U	T	M	U	T	M	U	T	M	U	T	M	U	T	M	U	T	M	U	T
10	0	104	5	16	153	1	16	124	1	17	106	4	182	101	1	22	103	0	19	101

a Including 68 *T. monococcum* ssp. *monococcum*, 9 *T. monococcum* ssp. *aegilopoides*, and 261 *T. monococcum* ssp. *boeoticum* lines.

b With exception of lines ID1122, ID393, ID1429, ID394, and ID1277.

c Average no. fragments in standard *T. monococcum* lines ID49, ID69, ID581, ID609 and ID1143.

d The definition of these lines is based on their topography in the PCA analysis of Figure 1.

e Average no. of fragments in standard *T. urartu* lines ID1364, ID1415, ID1438, ID1515, and ID1545.

The phylogenetic tree highlighting the genetic relationship within the group of *T. urartu* accessions (Figure S2) makes evident a clear association between genetic and geographical distances.

### Fertility in interspecific crosses

The results of the interspecific crosses between *T. urartu* and *T. monococcum* are presented in Table 2. All crosses with *T. urartu* lines used as female failed to produce viable F_2_ plants. When *T. m. monococcum* or *T. m. boeoticum* plants were pollinated by *T. urartu* pollen, a few F_1_ and F_2_ plants were obtained, particularly when the seed-bearing lines were wild *T. m. boeoticum* accessions. The percentage of fertile F_1_ seeds varied between 0 (ID396 × ID1277) and 4.5 (ID752 × ID1277). In the F_2_ generation the fertility increased up to 79% in some cases (ID758 × ID1122) and one family of the population B54 (from ID396 × ID1277) reached 89% fertility in the F_3_ generation. Interestingly, the ID1122 offspring had limited fertility in F_1_ (similar to other accessions); however, fertility in F_2_ generation was usually good, and greater than that of progenies derived from other *T. urartu* parents.

**Table 2:** Results of crosses among *T. urartu* (genome A^u^) and *T. m. monococcum* (genome A^m^) and fertility of the derived progenies

♀	♂	F_1_ Plants Grown					F_2_ Plants Grown			Further Generations
		No. Plants	No. Ears	No. Spikelets tested	Total No. of Seeds	% Fertility*^a^*	No. Plants	% Fertility*^a^*	No. Plants	% Fertility (Range)*^a^*
*T. m. monococcum*	*T. urartu*									
ID 396	ID 1122	5	53	<3000	3	<0.05	2		280 (F_3_)	0−84.5
									80 (F_3_)	8.5−89.0
ID 396	ID 1277	1	5	100	0	0				
L 118	ID 388	7	−	700	33	0.26−1.44	5		0–35	71 (S_1_BC_5_)
									0–23	48 (BC_6_)
*T. m. boeoticum*	*T. urartu*									
ID 752	ID 1122	2	9	274	14	0.8−4.1	6	29−77		
	ID 1277	2	22	200	11	1.0−4.5	7	5−29		
	ID 1391	2	13	200	7	0.5−3.0	1	4		
	ID 393	1	9	373	10	0−1.0	9	0.5−67		
ID 758	ID 1122	6	53	600	6	0−1.5	5	3−79		
	ID 1264	2	12	200	1	0−0.5	0			
	ID 1277	6	52	562	2	0−1.0	0			
*T. urartu*	*T. m. monococcum*									
ID 1391	ID 396	1	5	100	7	3.5	0			
*T. urartu*	*T. m. boeoticum*									
ID 1122	ID 752	1	2	58	0	0				
ID 1391	ID 758	1	9	100	0	0				
ID 393	ID 752	5	48	500	0	0				
ID 1264	ID 752	4	27	338	2	0 - 1	0			

a Based on the assumption of 2 florets/spikelet.

The hybrid between ID396 (*T. m. monococcum*) and ID1122 (*T. urartu*) gave rise to three F_2_ seeds, present in 53 F_1_ spikes. Two seeds produced partially fertile F_2_ plants, which generated the populations B53 (280 progenies) and B54 (80 progenies). Fertility and seed weight were investigated in successive segregating families (Figure S3). A substantial proportion of these families had sterile spikelets assigned to the fertility class 0.9–9%. The remaining B53 F_3_ progenies were characterized by an almost normal distribution, with a mode class of 40–49.9% fertility.

In the case of the 80 B54 progenies, the sterile spikelets were fewer compared with the B53 progenies and the fertility distribution was bimodal, having peaks in the 20–29.9% and 70–79.9% ranges. Seed weight was distributed normally in both populations. The modes and means of the two distributions were different: B53 had a mode class of 30-34.9 g and an average weight of 31.4 g per 1000 seeds, whereas B54 displayed a mode of 15-19.9 g and an average weight of 19.4 g per 1000 seeds.

The data support the conclusion that, although *T. monococcum* and *T. urartu* crosses produce mostly sterile F_1_ plants, it is still possible to recover a high degree of fertility in rare hybrid-derived progenies and to develop introgression lines.

### Recombination between A^m^ and A^u^ chromosomes

**Figure 2:**
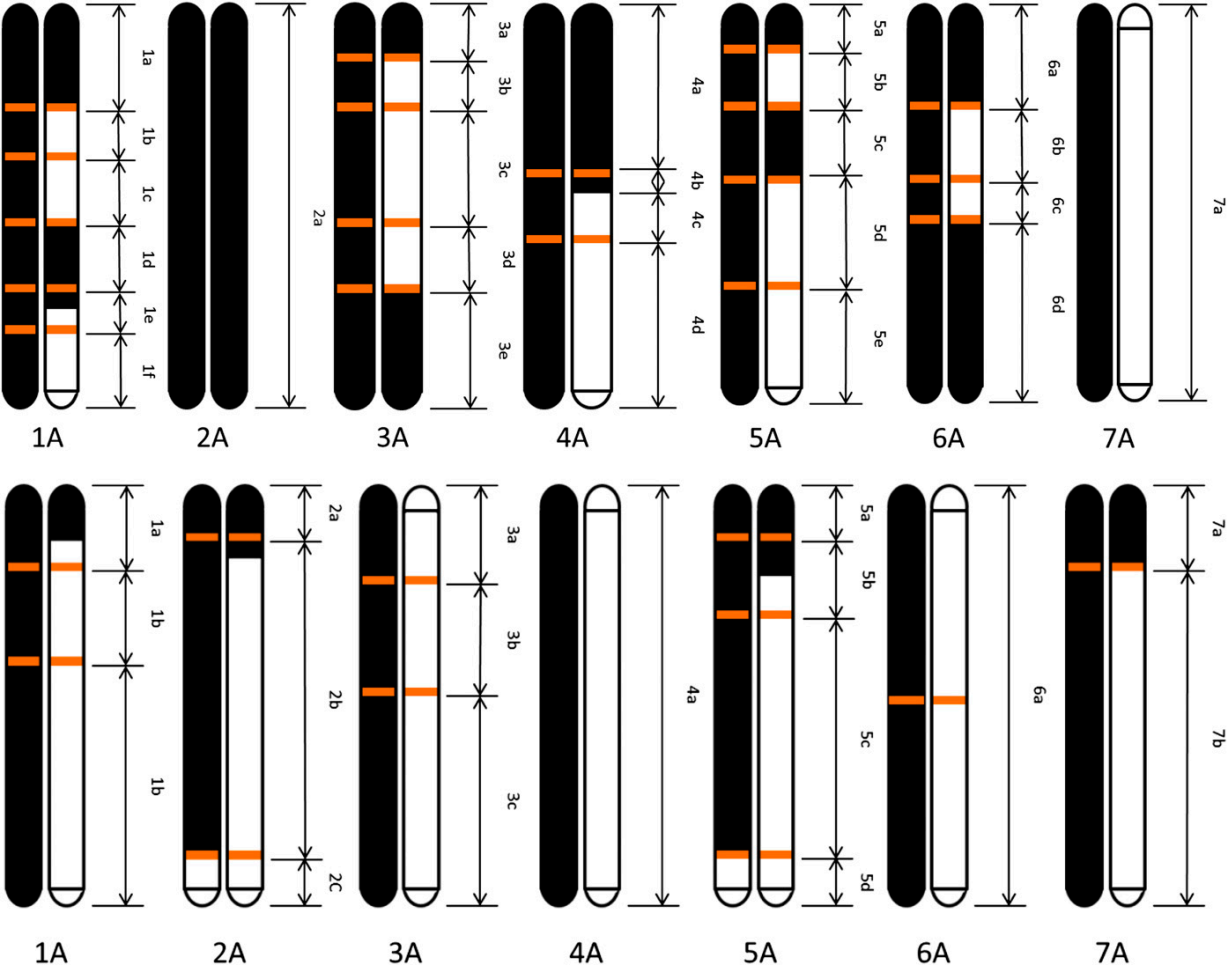
Reconstruction of the parental chromosome contribution to the F_2_ plants from which the populations B53 (above) and B54 (below) were developed. Parents *T. urartu* ID1122 and *T. monococcum* ID396 are in white and black, respectively. Double arrows indicate the borders of chromosome segments to which groups of amplified fragment-length polymorphisms were anchored based on the linkage map of *T. monococcum*. The chromosome position of the recombination sites detected in this analysis is shown as orange bars.

The genetic makeup, in terms of parental contribution, of the two F_2_ plants which originated the B53 and B54 populations of F_3_ plants is presented in Figure 2 and in File S1 (described in the section *Materials and Methods*). The details presented in the two maps support the following conclusions. In the B53 population, the chromosomal contribution of *T. monococcum* prevails. Chromosome 2 was inherited only from *T. monococcum*. Nine of 14 chromosomes did not present any recombination between A^m^ and A^u^ genomes. Chromosome 4 showed a recombination event, chromosomes 3 and 6 two events, and chromosomes 1 and 5 three events. In the B54 population, the chromosomal contribution of the two parental species was more balanced with six out of 14 chromosomes having one recombination event.

The finding of genetic recombination among A^m^ and A^u^ chromosomes was the basis to decide the development of introgression lines. This was carried out by means of a new crossing program using a line of *T. m. monococcum* with several positive agronomic traits as recurrent parent.

### Development of interspecific introgression lines

**Figure 3:**
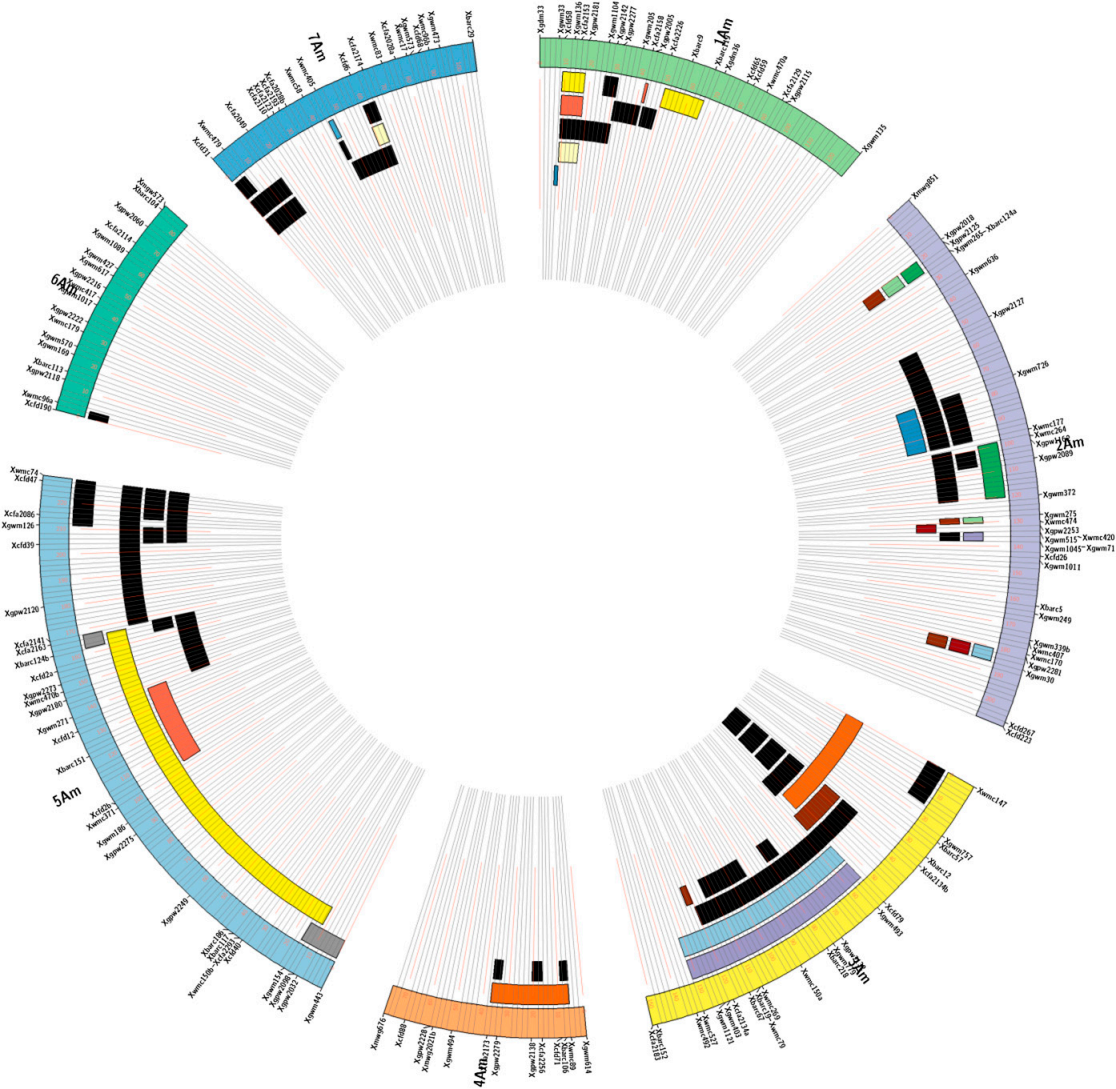
Linkage map of *T. monococcum* (outer circular segments) based on 121 F_2_ individuals of the ID 49 × ID 69 mapping population, and representation of the introgression lines of *T. urartu* ID388 in *T. monococcum* L118 anchored to linkage groups. For each of the seven linkage groups, the map positions of the corresponding molecular markers are reported. Black bars represent single chromosome segments of *T. urartu* detected in the introgression lines, while bars with the same color point out multiple chromosome segments of *T. urartu* detected in a single introgression line. Chromosome segments of *T. urartu* were anchored to ID 49 × ID 69 linkage map of einkorn when segregating in coupling together with specific chromosome A^m^ markers (see the section *Materials and Methods*). The recent sequencing of the *T. urartu* genome (Ling *et al.* 2013) will, in the future, allow a more precise definition of the recombination sites between A^u^ and A^m^ chromosomes.

The segregation of molecular markers concerning F_2_ plants derived from the cross ID69 × ID49, was processed by the JoinMap 4.0 program (Kyazma B.V., Wageningen, The Netherlands), to map markers used in the characterization of introgression lines (Figure 3). The obtained map contains 155 SSR marker loci without segregation distortion and distributed in seven linkage groups for a total of 984 cM, with an average value of one molecular marker every 6.35 cM. Linkage groups 1 to 7 include, respectively, 22, 28, 21, 13, 29, 16, and 19 SSR loci.

As described in the section *Materials and Methods*, the offsprings from the cross L118 × ID388 were backcrossed and self-fertilized for several generations. At each cycle the microsatellite markers with a known position on the ID69 × ID49 segregating population were used to select, in the background of the recurrent parent L118, plants carrying non redundant chromosome segments of *T. urartu*. Forty-six introgression lines (each harboring a single introgression chromosomal fragment of *T. urartu*) were isolated (Figure 3). The current state of development of our interspecific introgression lines is summarized in Table S3.

*T. urartu* introgression segments were anchored to the *T. monococcum* map, where they covered 580 of 984 cM: considering introgression lines available for each linkage group, the linkage groups 1−7 were respectively covered with *T. urartu* fragments of 57.1 cM (10 fragments; 44.6%), 96.6 cM (16 fragments; 46.4%), 131.9 cM (13 fragments; 87.1%), 36.7 cM (4 fragments; 48.4%), 229.4 cM (11 fragments; 100%), 2.9 cM (1 fragment; 3.4%), and 35.2 cM (8 fragments; 33.0%).

In the genetic map few *T. urartu* marker loci of a single introgression line appeared separated by large genetic intervals. For instance, *T. urartu* loci in introgression line 7183_5_1, despite several cycles of backcrossing and self-fertilization, still co-segregated without showing further recombination events. The same was observed for introgression line 7189_10_12 (LG1A^m^), 7177_16_4, 7178_3 and 7177_16_4 (LG2A^m^), and 7189_10_3 (LG5A^m^). One possibility is that the chromosomal region concerned is inverted in the two species. *T. urartu* loci of lines 71778_16_1 and 71778_16_1 mapped to different linkage groups, suggesting that in *T. urartu* duplicated chromosome blocks may exist.

**Figure 4:**
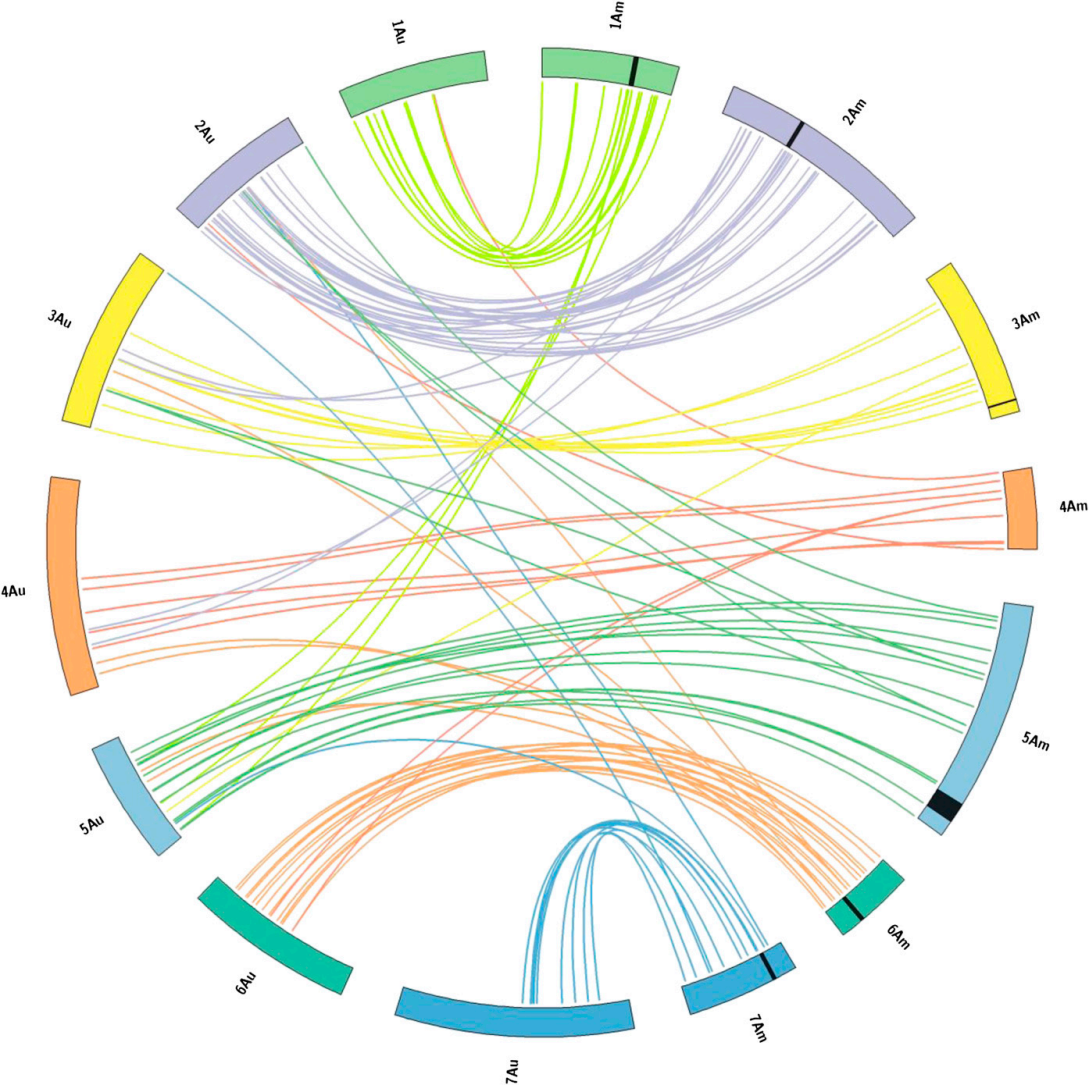
Comparative macrocolinearity relationships between A^u^ and A^m^ genomes. Homeologous chromosomes are reported as specular circular segments with the same color. Chromosomes of the A^m^ and A^u^ genomes are specified (from 1 to 7 A^m^ or A^u^). Lines connect map position of microsatellite loci in the A^m^ genome (left) to *T. urartu* paralogous (right). Lines joining A^m^ and A^u^ chromosomes with different colors point to loci mapping in nonhomeologous linkage groups in *T. monococcum* and *T. urartu*. Lines joining A^m^ and A^u^ chromosomes with the same color evidence microsatellites loci mapping in *T. monococcum* and *T. urartu* in homeologous linkage groups.

The results presented in Figure 4 point to a high degree of conservation of marker order between A^u^ and A^m^ chromosomes. Exceptions concerned three chromosome 1 *T. monococcum* markers mapping to *T. urartu* chromosome 5, four *T. monococcum* chromosome 2 markers located on *T. urartu* chromosomes 3 and 4, and other 16 markers of markers of *T. monococcum* chromosomes 3, 4, 5, 6 and 7 mapping to different *T. urartu* chromosomes.

### A test of the variation among introgression lines

Significant differences between *T. urartu* and *T. monococcum* parents were evident for all compounds measured, with the exception of α- + β-carotene. Lutein, α- + β-carotene, β-cryptoxanthin, and β-tocotrienol content showed significant transgressive phenotypes with higher levels in different introgression lines compared to both parents (Table 3). The comparative analysis of zinc, iron and calcium content in kernels of the introgression lines revealed a transgressive phenotype in introgression line 7183_1_1 for zinc content, while calcium and iron contents did not exhibit significant variations.

**Table 3:** Contents of α- + β-carotenes, β-cryptoxanthin, zeaxanthin, lutein, α-tocopherol, α-tocotrienol, β-tocopherol, β-tocopherol, zinc, calcium, and iron in donor (ID388) and host (L118) parents and in 28 interspecific introgression lines

Compound or Microelement, mg/kg	Average Value		*P* Value of Difference Between Parents	No. of Interspecific Introgression Lines (of 28) with Contents Significantly*^a^* Greater Than the Best Parent
	L118	ID388		
α- + β-carotenes	0.209	0.220	> 0.05	17
β-cryptoxanthin	0.046	0.026	≤ 0.001	6
Lutein	4.039	4.452	≤ 0.05	16
Zeaxanthin	0.182	0.384	≤ 0.001	0
α-tocopherol	8.372	13.286	≤ 0.001	0
α-tocotrienol	8.556	16.406	≤ 0.001	0
β-tocopherol	3.000	4.513	≤ 0.01	0
β-tocotrienol	32.566	38.129	≤ 0.01	3
Zn	0.730	2.235	≤ 0.001	1
Fe	0.470	0.505	≤ 0.05	0
Ca	2.555	3.710	≤ 0.01	0

a Based on *t*-test; see the section *Materials and Methods*.

## Discussion

The fertility of interspecific hybrids between *T. monococcum* and *T. urartu* is associated with the directionality of the cross. When *T. urartu* was the pollen acceptor, F_1_ hybrid plants were not obtained. In contrast, *T. urartu* pollen fertilizing *T. monococcum* eggs yielded almost sterile F_1_ hybrid plants which generated rare fertile progenies (Table 2). The results parallel those of Dhaliwal (1977b), Sharma and Waines (1981), Lucas and Jahier (1988), and Navruzbekov (1989) although these authors considered only the F_1_ hybrid generation. As direct and reciprocal hybrids are *bona fide* identical at the nuclear level, different levels of F_1_ fertility depending on cross directionality could be linked to epigenetic factors. Such factors also act on plant vigor (Table 1). The comparative effect of reciprocal crosses has been extensively studied in maize, where significantly different vigor and phenotypes were associated to reciprocal crosses and 4000 expression quantitative trait loci were mapped (Swanson-Wagner *et al.* 2009). In maize, different transcript accumulation is consistent with gene expression in the hybrid being regulated by the paternally transmitted allele, supporting the conclusion that a widespread parental imprinting contributes to gene expression under hybrid conditions (Swanson-Wagner *et al.* 2009). In addition, it is known that in general fertility depends on the interaction between nuclear and mitochondrial genomes (Frank 1989; Chase 2007). It is possible that these interactions could also play a role in the viability and fertility of reciprocal interspecific hybrids between *T. urartu* and *T. monococcum*.

A relevant finding reported in this paper is the existence of transition forms that bridge the genetic gap between the two A-genome species. Contrary to this finding, a clear-cut split between *T. urartu* and *T. monococcum* was consistently recorded by several authors (Smith-Huerta *et al.* 1989; Vierling and Nguyen 1992; Castagna *et al.* 1997; Mizumoto *et al.* 2002; Sasanuma *et al.* 2002; Brandolini *et al.* 2006), and rare *T. urartu* accessions spotted within the germplasm collections of *T. monococcum* were dismissed as misclassified samples (Hammer *et al.* 2000). The *T. urartu* accessions characterized in their genome by the presence of *T. monococcum* marker alleles apparently influence the fertility of *T. monococcum* × *T. urartu* hybrid plants (the case of crosses involving lines 1122, 1277, 393). In our interspecific F_3_ populations, peaks of fertility as high as 84.5 or 89% were recorded. Therefore, it is tempting to speculate that the correlation between sterility and genetic distance alone may be sufficient to explain the survival and prevalence of genotypes more and more similar to the two parental species (Oka and Chang 1961; Nolte and Tautz 2010). This hypothesis can be properly tested based on interspecific introgression lines. Any possible effects of the *T. urartu* DNA on plant fertility can, in fact, be attributed to specific chromosome fragments of *T. urartu*.

Given its ample molecular and phenotypic diversity (Hegde and Waines 1997; Kilian *et al.* 2007), *T. monococcum* is an attractive gene donor to polyploid wheats. In this respect, the availability of introgression lines represents an important addition to the prebreeding value of crosses targeted to improve common and durum wheat, particularly when, as in our case, the use of introgression lines in breeding schemes can be assisted by molecular markers (Eshed and Zamir 1995).

To date introgression line populations have been developed in a number of crop plants, in particular introgressing wild relative chromosome segments in elite varieties. In this study introgression lines were constructed starting from two species which have a low level of sexual compatibility. This is one of the first reports in which such phylogenetically distant species have been used to develop interspecific introgression lines. To this end, a large number of plant lines were subjected to molecular fingerprinting; nevertheless, only 46 introgression lines were isolated and anchored to 580 of 984 cM of the linkage map of *T. monococcum*. Introgression lines, however, were not anchored in the remaining 404 cM: two main hypotheses can be proposed to account for this observation.

1. Reconstruction of the chromosomal organization of the gametes extracted from *T. monococcum* and *T. urartu* helped in revealing severe segregation distortions that ultimately did not allow some loci to be transmitted to the offspring. Thus, segregation distortion linked to gametic selection in hybrids seems to be a major player. This interpretation is consistent with the finding that most *T. urartu* chromosome segments not anchored in the ID49 × ID69 genetic map were not detected in the BC_1_ backcross population. Given that the number of F_1_ plants backcrossed to the recurrent parent *T. monococcum* L118 was sufficiently large, the failure to observe such *T. urartu* loci can reasonably be attributed to segregation distortion, leading to elimination of specific chromosome fragments.
2. Chromosome pairing may have played a key role, as shown by the macrocolinearity analysis revealing the presence of chromosome translocations between *T. urartu* and *T. monococcum* genomes. Linkage group 1A^m^ was previously reported to have undergone large chromosomal rearrangements compared to linkage group 1A^u^ (Dubcovsky *et al.* 1996). In our analysis, chromosomal rearrangements were demonstrated not only for linkage group 1A^m^, but for all seven chromosomes that underwent different degrees of macrocolinearity erosion. These rearrangements are supposed to play a central role in segregation distortion. In pioneering studies on *T. monococcum* and *T. urartu* hybrids, a correct chromosome pairing was reported (Dhaliwal 1977a). Nevertheless, our findings suggest that many of the chromosomal rearrangements between these two species may significantly hinder perfect matching of chromosomes, thus limiting the recombination to specific genomic regions in such hybrids.

The phenotypic analysis carried out on kernels of a subset of 28 introgression lines revealed good variability for the traits investigated. These are already indicative that several *T. urartu* chromosome segments affect relevant quality traits, implying that QTL for these traits could later be precisely described and associated to specific marker alleles. Although preliminary, the test based on 28 introgression lines demonstrates that the introgression lines of *T. urartu* ID 388 *in T. monococcum* L118 have potential breeding applications.

In conclusion, the principal purpose of the experiments described in this paper was the creation of *T. urartu* introgression lines in an agronomically improved *T. monococcum* genotype. To arrive at the final list of 46 lines (described in Table S3), it was necessary to carry out several preliminary experiments addressing the following: the existence of natural genetic variability pointing to the presence (in primary habitat populations) of intermediate forms between the two species, the possibility of obtaining progenies with at least a certain degree of fertility from *T. monococcum* × *T. urartu* crosses, the existence of pairing and genetic exchange between couples of *T. monococcum*, and *T. urartu* chromosomes and the choice of molecular markers specific for the A genome of wheat to create a genetic map of *T. monococcum* to which anchor fragments of *T. urartu* chromosomes. The 46 introgression lines are now available for further experiments.

## Supplementary Material

Supporting Information
