# Supplementary material for: Crossability of Triticum urartu and Triticum monococcum Wheats, Homoeologous Recombination, and Description of a Panel of Interspecific Introgression Lines
Source: G3 (Bethesda). 2014 Aug 21;4(10):1931–41. doi: 10.1534/g3.114.013623 (PMC4199699; doi:10.1534/g3.114.013623)
Supplement: Supporting Information [file supp_4_10_1931__index.html]

Crossability of Triticum urartu and Triticum monococcum Wheats, Homoeologous Recombination, and Description of a Panel of Interspecific Introgression Lines — Supporting Information 

# Crossability of *Triticum urartu* and *Triticum monococcum* Wheats, Homoeologous Recombination, and Description of a Panel of Interspecific Introgression Lines

## Supporting Information for Fricano *et al.*, 2014

**Files in this Data Supplement:**

- Supporting Information - Figures S1-S3, Files S1-S3, and Tables S1-S4 (PDF, 277 KB)
- Figure S1 - Geographic distribution and number of AFLP-fingerprinted *Triticum monococcum* ssp. *boeoticum* (red circles) and *Triticum urartu* (green circles) accessions. (PDF, 171 KB)
- Figure S2 - Principal Coordinates Analysis of the *T. urartu* wheat accessions. (PDF, 174 KB)
- Figure S3 - Fertility (%) and 1000 seeds weight (g) distribution in the B53 (n= 268) and B54 (n= 80) populations. (PDF, 136 KB)
- Table S2 - Sequences of probes used for amplifying GPW microsatellites. (PDF, 121 KB)
- Table S3 - List of the introgression lines created in this study. (PDF, 123 KB)
- Table S4 - Supplementary information for Figure 2. (PDF, 148 KB)
- Table S1 - Diploid wheat accessions considered for AFLP fingerprinting. (.xls, 118 KB)
- File S1 - Mapping data of the diploid wheat lines. (.xlsx, 441 KB)
- File S2 - Mapping data of the B53 recombinant population. (.xls, 354 KB)
- File S3 - Mapping data of the B54 recombinant population. (.xls, 72 KB)
