# Supplementary material for: Crossability of Triticum urartu and Triticum monococcum Wheats, Homoeologous Recombination, and Description of a Panel of Interspecific Introgression Lines
Source: G3 (Bethesda). 2014 Aug 21;4(10):1931–41. doi: 10.1534/g3.114.013623 (PMC4199699; doi:10.1534/g3.114.013623)
Supplement: Supporting Information [file supp_g3.114.013623_013623SI.pdf]

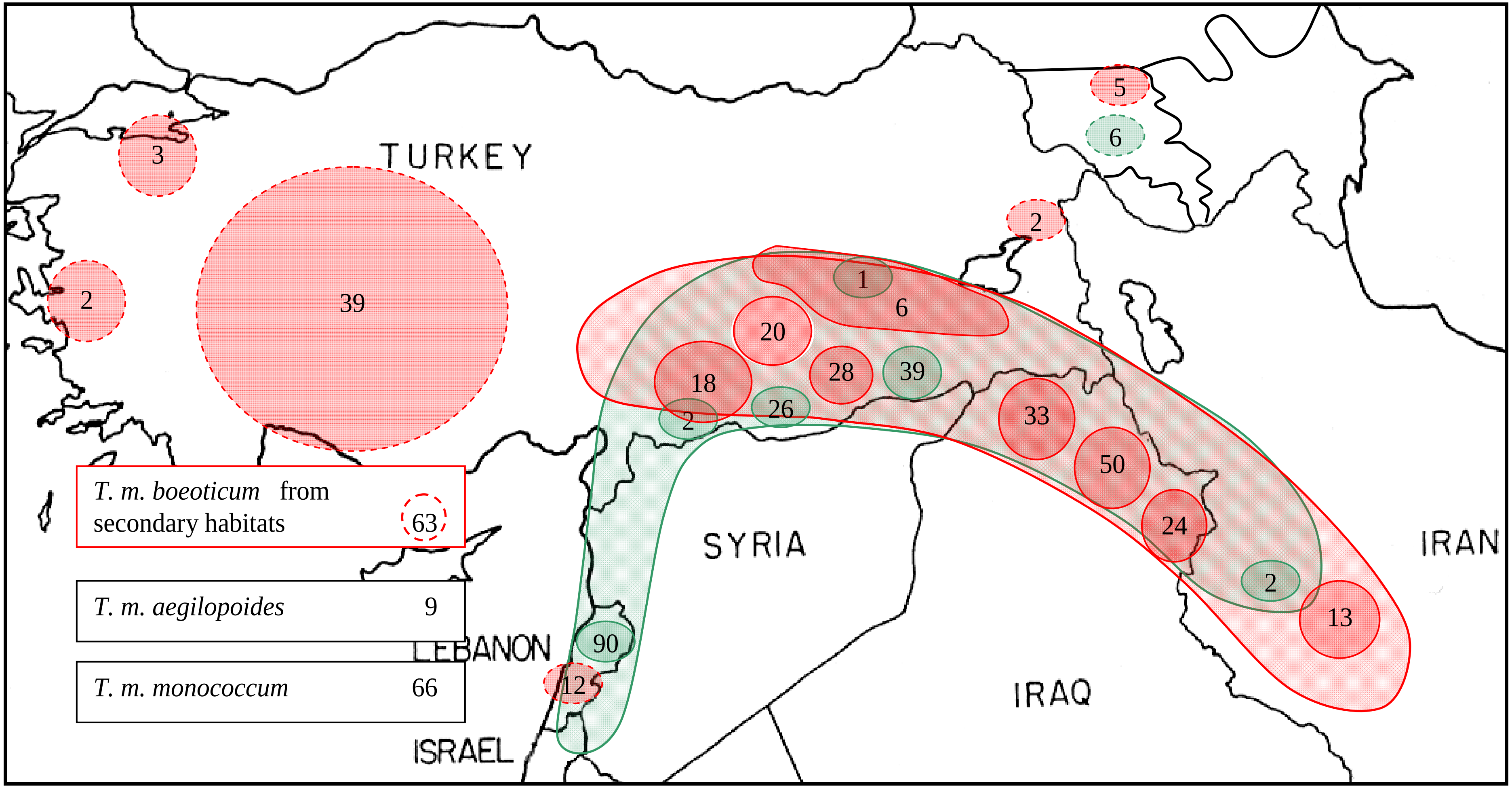

Figure S1 Geographic distribution and number of AFLP-fingerprinted *Triticum monococcum* ssp. *boeoticum* (red circles) and *Triticum urartu* (green circles) accessions. The red- and the green-delimited areas correspond to the primary habitats of *T. m.* subsp. *boeoticum* (Heun et al., 1997) and of *T. urartu* (see Valkoun et al., 1998), respectively. The number of *T. m.* ssp. *boeoticum* from secondary habitats, as well as ssp.*monococcum* and ssp. *aegilopoides* acccessions, is also reported.

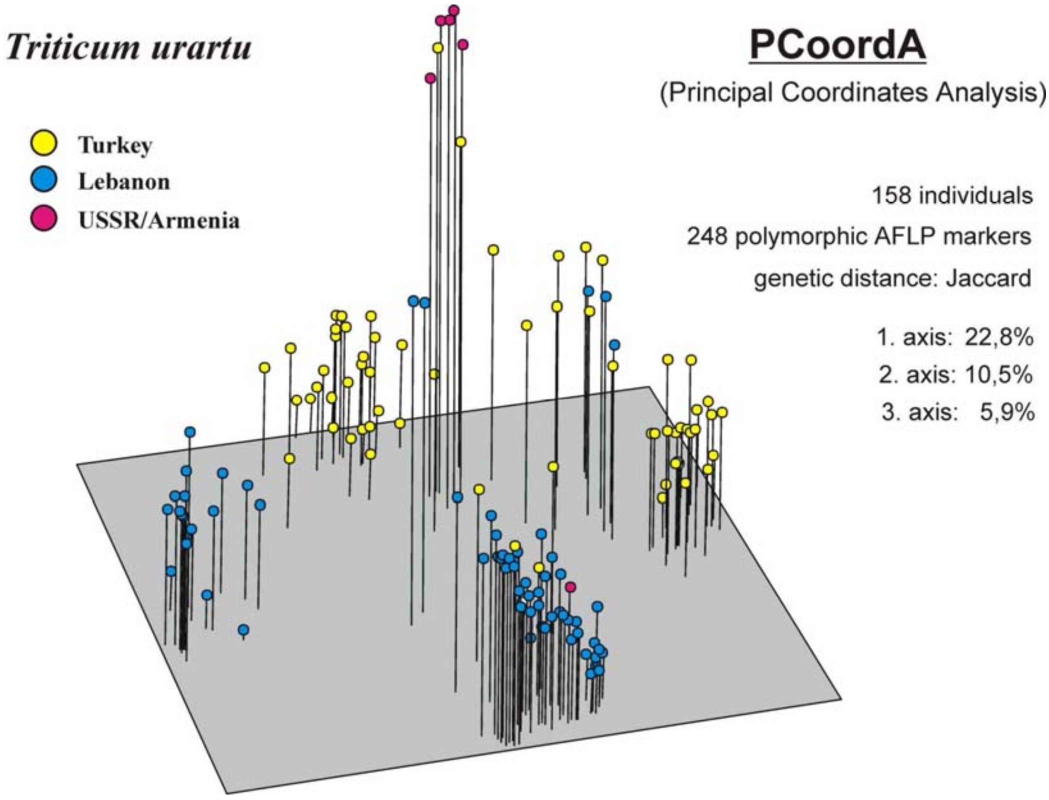

Figure S2 Principal Coordinates Analysis of the *T. urartu* wheat accessions. In this analysis, the lines molecularly intermediate between *T. urartu* and *T. monococcum* have not been considered.

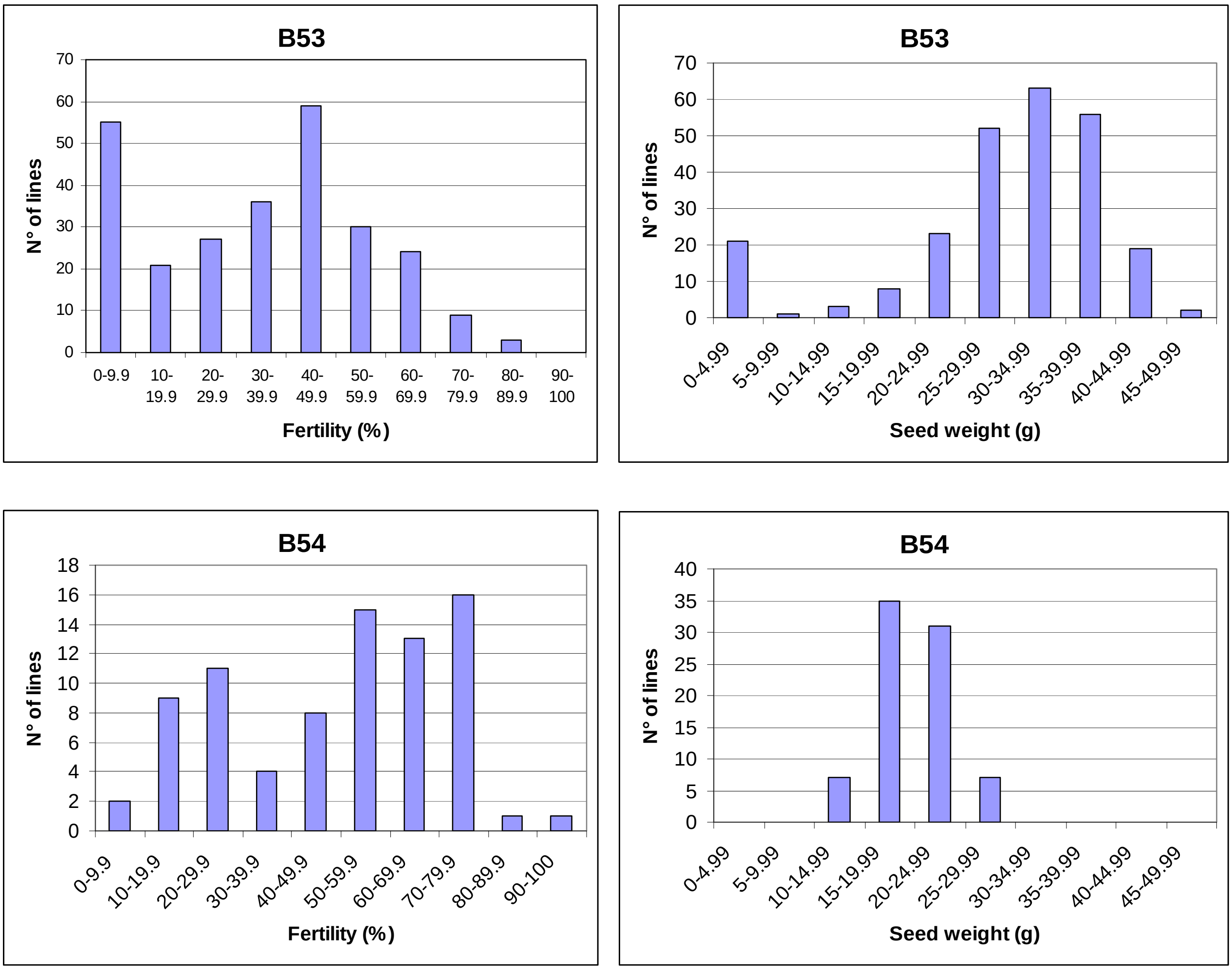

Figure S3 Fertility (%) and 1000 seeds weight (g) distribution in the B53 (n= 268) and B54 (n= 80) populations

Files S1-S3

Available for download as Excel files at http://www.g3journal.org/lookup/suppl/doi:10.1534/g3.114.013623/-/DC1

File S1 Mapping data of the diploid wheat lines

File S2 Mapping data of the B53 recombinant population

File S3 Mapping data of the B54 recombinant population

Table S1 Diploid wheat accessions considered for AFLP fingerprinting.

Available for download as an Excel file at http://www.g3journal.org/lookup/suppl/doi:10.1534/g3.114.013623/-/DC1

Table S2 Sequences of probes used for amplifying GPW microsatellites.

SSR	Sequence primer Forward (5'—3')	Sequence primer reverse (5'—3')
gpw_2006	ATGAGAAGGGGGTCAGGATT	ACATGTTCAGCCCAGGAGAC
gpw_2010	ACCCATTGCCTTTTCTTTTT	TCTGTTGATGATCCGTCCAA
gpw_2018	ATGTAGGCAGAGCACACACG	AGTCGATGAAAGGCAGCATC
gpw_2029	TAAAGCTAAACACGACGGGG	CACCGCGAACGAATTAAAAC
gpw_2032	CCTGGAAGAATAGACGTGCC	CAAGATGGGGCAGAAGATGT
gpw_2069	AGGAGAAGGCGTAAGAACCC	GGCAAGCTGGTCCTGTGTAT
gpw_2080	ATCGCATGTAACCTGCACAA	CCTTTAATCGATTGCTCGGA
gpw_2098	ACACACCCGCAAAATAATCT	TGACGCCACATAGGTCAATC
gpw_2109	TATATTGTCCACGGGGCTTC	TGGTGGAGAGTCTTGCACTG
gpw_2111	AAATTTTTGTCTGCCGGCTT	CTTGTGCGTTGAGAGTTGGA
gpw_2115	TTACAAGGCCGTAAATTGCC	TGCTTGCTGACCACTGAATC
gpw_2117	TGGCCTGAAATCTTAGCCTG	CAAGAATGCGATAAGATGGGA
gpw_2125	GGATGGGAAATGTTTGGATG	AAAATCAAACGGCAACTTGG
gpw_2127	GACAACACCGATCCGTCAC	TGTCCATGCGTTCTATTCCA
gpw_2132	TCCCGCAAATATGTGGCTAT	TATGTTGCATTGTGGTGGCT
gpw_2138	ATAGGAGGACTCCTGGGCTC	TTGCCTCAACTAGATCGCCT
gpw_2139	TGTTAACCCAGTTTCCTTATGC	ACACTGATGCATCCCACAAA
gpw_2140	GTCCACGTGCTAGGGAGGTA	ACATGCCCTAAGCTGCCCCA
gpw_2142	ACAACTGCTGCAGCTCCTTC	GATTAATTAAGCCAGGGACCG
gpw_2160	ATTTACGGCTCGACCACTCA	ACTGGAAAGGGGCGCAAGC
gpw_2166	GCCCCTGACATATTATTCACTGT	AAACTGGATGGTTGCATTCC
gpw_2169	GCCAGGCCATCAGTAAATTC	AATGGGCACAATTTGAGAGC
gpw_2216	ACGAGGAATTGCATCCTAGC	CAAAGTAGAAATTTATGCGCGA
gpw_2222	TCTCAGGAGCTAGCAGCACA	CTTCTGCCGATACATCCCAT
gpw_2228	TGTAGCTTCTGCATCACCAAA	CAAACTTGCGAGCTGCATTA
gpw_2229	CTGCGTGCTGCTCCTAATTT	CTCCACCGTGTCCTGGATAG
gpw_2237	CTTTGCTTGCGGTAGGAGAC	TGATCTATCAGGGTGAGCCC
gpw_2239	CAACCATATGCCCAGGAGAC	TGTTGCTGTCTGAAACAGGG
gpw_2243	GGGCAATCTGTTGGATCTGT	CCACTTCGCTGCTGATGTAA
gpw_2250	AGCCATAGATGGCCCTACCT	CACTCAATGGCAGGTCCTTT
gpw_2253	TGAGGAGAGGGGATATACGG	TTTGCGAAATCTTATTGCCC
gpw_2258	ATATAGGGCCGATGTGTGGA	GGTCAGCAAAGTCAGCCTT
gpw_2260	CATCTCTACCCGATCCCTCA	ACGCCGGTCTATTGAAAGTG
gpw_2264	TTGCTTTCCCAAATTGTGCT	GGCATTGAGAATCCAAGCAT
gpw_2266	TTTTTGCCCACACGGC	CGTGGAGGTGTCGACCTAAT
gpw_2269	CACATCAACAGGTCCTCTTCTA	CTAGCTGGTGGTGGTCTTGG
gpw_2270	GAGGTCGTTGAAGGGAAGG	ATCGGACGGCCTGAGTTATA
gpw_2275	CTGCTGAACGTTTGGAGGAT	GGCCGTCCTTTAGCTTTTGT
gpw_2276	ATAGGGTTCTTCTGTGCCCC	ACCCACAGTTGAACTTGGG
gpw_2277	TCAGAAGAGCGATGAGATAGAAA	GCCATTTTTAGGGCTCAGTG
gpw_2281	TCATCATGGTATGAGCGTGG	ACAAGCATTCCAATTTTGCC
gpw_2283	CTCTGTCACAACGAGCTGGA	AATGGGCTTCAGATGTCCTG
gpw_2297	TCGGAGAACCAAACTGATCC	GACTAACCACTGGGAGTCGC
gpw_2302	GCTTCACATCATAGTGTGGATAAGA	AAGCACCTCCCATGCATATC
gpw_2308	GGAGGAACCGAATCCAGAGT	GAGGCCGATCACATAAAGGA
gpw_2311	CCAAAAGTGGTGGGATCAAT	TGCAAGAACAGCTTACCGTC
gpw_2323	AGAAGTTGGCTTCCGCTTTC	AGTTGAAGATGGCCCAGATG
gpw_2328	ATCCCAACAAACACACTACCG	TTGTCTCCATGACTATGTGGG
gpw_2331	GCGGGCTCAATATTGCTAGT	GCATGGCTGAGGCTCAAGTA
gpw_2335	TTTGCAGTTGCCACAAAAGT	TGTTTTGTCTCACAGGCTGC

Table S3 List of the introgression lines created in this study. For each introgression line the left and right markers delimiting the chromosome segments of *T. urartu* are reported. The intervals where each chromosome segment is anchored in the linkage map of *T. monococcum* are also reported.

Linkage Group	Left Marker	Right Marker	Interval (cM)	Zygosity	IL Name
4	Xgpw2279	Xcfa2173	3.59	Hetero	7197-16-9
4	Xcfa2256	Xgpw2138	4.08	Hetero	7197-16-8
3	Xwmc150a	Xbarc67	21.4	Hetero	7197-16-7
3	Xgpw2132	Xbarc218	6.5	Hetero	7197-16-6
2	Xgwm515	Xgwm1045	3.62	Hetero	7197-16-4
2	Xgpw1162	Xgpw2089	4.0	Homo	7197-16-3
1	Xgpw2005	Xcfa2226	5.8	Hetero	7197-16-2
7	Xwmc405	Xcfa2174	18.94	Hetero	7197-16-12
7	Xcfd31	Xcfa2049	16.47	Hetero	7197-16-11
7	Xcfd31	Xcfa2049	16.47	Hetero	7197-16-10
1	Xgpw2277	Xcfa2158	10.93	Hetero	7197-16-1
7	Xcfd31	Xwmc479	5.19	Homo	7189-8-8
1	Xcfd58	Xgpw2277	22.13	Homo	7189-2-2
5	Xcfa2086	Xwmc74	14.46	Homo	7189-10-6
1	Xgwm1104	Xgpw2277	5.38	Homo	7189-10-4
5	Xgwm443	Xgwm154	15.63	Homo	7189-10-3
5	Xbarc124b	Xcfa2141	5.32	Homo	7189-10-3
7	Xcfd6	Xcfa2174	4.96	Homo	7189-10-14
5	Xgwm126	Xwmc74	18.27	Homo	7189-10-13
1	Xcfd58	Xgpw2181	9.07	Homo	7189-10-12
1	Xbarc9	Xbarc9	16.12	Homo	7189-10-12
5	Xgpw2098	Xcfa2163	155.33	Homo	7189-10-12
5	Xcfa2141	Xwmc74	61.71	Homo	7188-1-2
3	Xcfa2134b	Xcfa2134a	84.9	Homo	7188-1-1
5	Xcfd39	Xwmc74	25.53	Homo	7183-8-2
1	Xcfd58	Xgpw2181	9.07	Homo	7183-5-1
1	Xcfa2158	Xgpw2005	1.1	Homo	7183-5-1
5	Xcfd2b	Xgwm271	35.61	Homo	7183-5-1
6	Xcfd190	Xwmc96a	2.88	Homo	7183-3-1
3	Xwmc147	Xcfd79	53.9	Homo	7183-2-2
4	Xwmc89	Xcfa2173	31.11	Homo	7183-2-2
3	Xcfa2134b	Xcfa2134b	14.7	Homo	7183-2-1
3	Xcfa2134b	Xcfa2134b	14.7	Homo	7183-1-2
3	Xwmc147	Xwmc147	15.5	Homo	7183-1-1
3	Xcfa2134b	Xcfa2134b	14.7	Homo	7181-1-2
7	Xwmc405	Xwmc405	2.0	Homo	7180-3-4
5	Xgwm271	Xcfa2141	28.91	Homo	7179-3-3
5	Xbarc124b	Xcfa2141	5.32	Homo	7179-3-2
5	Xcfd39	Xgwm126	7.26	Homo	7179-1-4
1	Xgwm33	Xcfd58	1.74	Homo	7178-6-1
2	Xgwm726	Xwmc177	20.74	Homo	7178-6-1
2	Xgwm726	Xwmc177	20.74	Homo	7178-4-1
2	Xgpw2125	Xbarc124a	5.48	Homo	7178-3-1
	Xwmc264	Xgwm372	21.98	Homo	7178-3-1
1	Xcfd58	Xgpw2181	9.07	Homo	7178-1-1

7	Xcfd6	Xcfa2174	4.96	Homo	7178-1-1
2	Xgpw2127	Xwmc177	43.94	Homo	7177-9-1
3	Xcfa2134b	Xgwm493	21.7	Homo	7177-16-6
2	Xgwm515	Xgwm1045	3.62	Hetero	7177-16-5
3	Xcfd79	Xwmc527	78.0	Homo	7177-16-5
2	Xgpw2125	Xbarc124a	5.48	Homo	7177-16-4
2	Xgwm275	Xwmc474	2.56	Homo	7177-16-4
2	Xgpw2281	Xgwm30	5.09	Homo	7177-16-3
3	Xcfd79	Xwmc527	78.0	Homo	7177-16-3
2	Xgpw2125	Xbarc124a	5.48	Homo	7177-16-1
2	Xgwm275	Xwmc474	2.56	Homo	7177-16-1
2	Xgpw2281	Xgwm30	5.09	Homo	7177-16-1
3	Xcfa2134b	Xgwm493	21.7	Homo	7177-16-1
3	Xcfa2134a	Xgwm1121	3.1	Homo	7177-16-1
2	Xwmc474	Xgwm515	3.82	Homo	7177-15-3
2	Xgpw2281	Xgwm30	5.09	Homo	7177-15-3
7	Xwmc405	Xwmc405	2.0	Homo	7176-11-1
4	Xwmc89	Xcfd71	3.26	Homo	7138-5-2

Table S4 Supplementary information for Figure 2

B53

1a: 7 bands homozygous mono (m404013, m355907, m366206, m376005, m326003, m364018, m376103)
1b: 6 bands from urartu in 31.1cM (u413346, u413836, u4038e, u413848, u404028, u373829*)*
1c: 3 bands from mono in 28.6 cM (m403255, m356001, m413851)
1d: 6 bands homozygous mono (m356006, m356104, m363625, m403212d, m4133b, m423301)
1e: 1 band from mono, 1 band from urartu in 6.4 cM (u364028, m423330); m and u bands in COUPLING
1f: 4 bands from mono in 26.8 cM + 4 bands from urartu in 29.2 cM (m374026, m403251, u413343, m423229, m403215f, u403846, u424040, u403250); m and u bands in REPULSION
2a: 10 bands homozygous mono (m363818, m374015, m356108, m413812b, m374019, m364009, m414014, m373807, m376109, m423321)
3a: 1band homozygous mono (m403224)
3b: cluster of 3 bands from mono in 10.8 cM (m404025, m373820, m424041)
3c: cluster of 9 bands from urartu in 18.9 and 5 bands from mono in 11.8 cM (u413340, u404032, u403851, u413842, u414030, u373821, u413344, u363637, u363815, u373822, m403259, m374034, m413232, m414036, m404026)
3d: 1 band from mono and cluster of 6 bands from urartu in 1.9 cM (m423314, u373337, u363642, u363635, u363636, u423846, u424045); m and u bands in REPULSION
3e: 3 bands homozygous mono (m326101, m326112, m423319)
4a: 6 bands homozygous mono (m404017, m374806, m4240a, m373316, m374010, m403225)
4b: 3 bands in 12.3 cM, 2 from urartu, 1 from mono (u424013, u404003, m403208); m and u bands in COUPLING
4c: 7 bands from mono in 18.7 cM (m423234, m413838, m373824, m373332, m374024, m413234, m413204)
5a: 1 band homozygous mono (m403835)
5b: 3 bands from mono in 12.5 cM + 2 bands from urartu in 4.6 cM (m364029, m424034, m404029, u404028, u373829*)*
5c: 7 bands homozygous mono (m356113, m413815, m355902, m413328, m363819, m373211, m373304)
5d: cluster of 12 bands from urartu in 10.4 cM + 4 bands from mono in 15.5 cM (u413837, u373342, u374032, u413342, u424037, u423844, u374027, u374028, u374031, u403262, u403843, u363633, m364030, m356109, m364024, m363632)
5e: 3 bands from mono in 8.9 cM + 3 bands from urartu in 12.4 cM (m326008, m413833, m413846, u413839, u363643, u374029)
6a: 5 bands homozygous mono (m363805, m373208, m363812, m3733b, m403217)
6b: 12 bands from mono in 63.2 cM + 4 bands from urartu in 20.7 cM (m424003, m373334, m366212, m364027, m364031, m326105, m374035, m423328, m404020, m403254, m413224, m414037, u423802, u424046, u374036, u414031)
6c: 2 bands from mono in 21 cM (m326111, m413306)
6d: 5 bands homozygous mono (m374808, m403818, m403831, m326006, m413814d)
7a: 2 bands from mono in 8.2 cM (m423806, m403247)
7b: 5 bands in from urartu in 37.9cM (u403841, u373828, u424039u413235, u364035)

B54

1a: 1 band from urartu and 2 bands from mono in 24 cM (m413819, m403836, m424030) IN REPULSION
1b: 3 bands from mono in 22 cM (m364018, m363625, m4133b)
1c: 1 band from urartu (m363629)
2a: 1 band homozygous from mono (m374015)
2b: 2 bands from urartu and 6 bands from mono in 50 cM (m374014, m364009, m403809, u3738b, u364001, m373807, m374003, m403821) IN REPULSION
2c: 1 band homozygous from urartu (m413818)
3a: 2 bands from urartu in 29 cM (m364001, m364004)
3b: 1 band from mono (m3738a)
3c: 2 bands from urartu in 9 cM (u413310, m4133a)
3d: 5 bands from urartu in 43 cM (u413304, u414801, u413307, u373301, u374008)
3e: 1 band from urartu (u413314)
4a: 8 bands from mono in 35 cM (m404017, u364004, m4240a, m374010, u364007, m373316, m364012, m413334)
4b: 1 band from mono (m374024)
5a: 2 bands homozygous from mono in 38 cM (m403835, m424034)
5b: 1 band from mono and 1 band from uraru in 17 cM (m4138c, m4038b) IN REPULSION
5c: 1 band from mono (m373304)
5d: 11 bands from urartu in 48 cM (u373806, u403808, u373809, u413801, u413805, u373302, u374004, u413303, m404006, u373813, u373802)
5e: 1 band from mono (m413326)
5f: 1 band homozygous from urartu (m413825)
6a: 1 band from mono (m3738b)
6b: 7 bands from mono in 44 cM (m4038c, u41339, u40389, m413820, m4038a, u363605, m413306)
6c: 4 bands from mono in 52 cM (u40387, m414806, u373810, u364008)
7a: 1 band homozygous from mono (m414810)
7b: 3 bands from mono in 19 cM (m3740a, m423806, u37383)

* Assigned to both 1b and 5b groups of B53 with a LOD > 3.0; preferentially assigned to group 1b with a LOD > 4.0
