# Supplementary material for: Crossability of Triticum urartu and Triticum monococcum Wheats, Homoeologous Recombination, and Description of a Panel of Interspecific Introgression Lines
Source: G3 (Bethesda). 2014 Aug 21;4(10):1931–41. doi: 10.1534/g3.114.013623 (PMC4199699; doi:10.1534/g3.114.013623)
Supplement: Supporting Information [file supp_g3.114.013623_FigureS2.pdf]

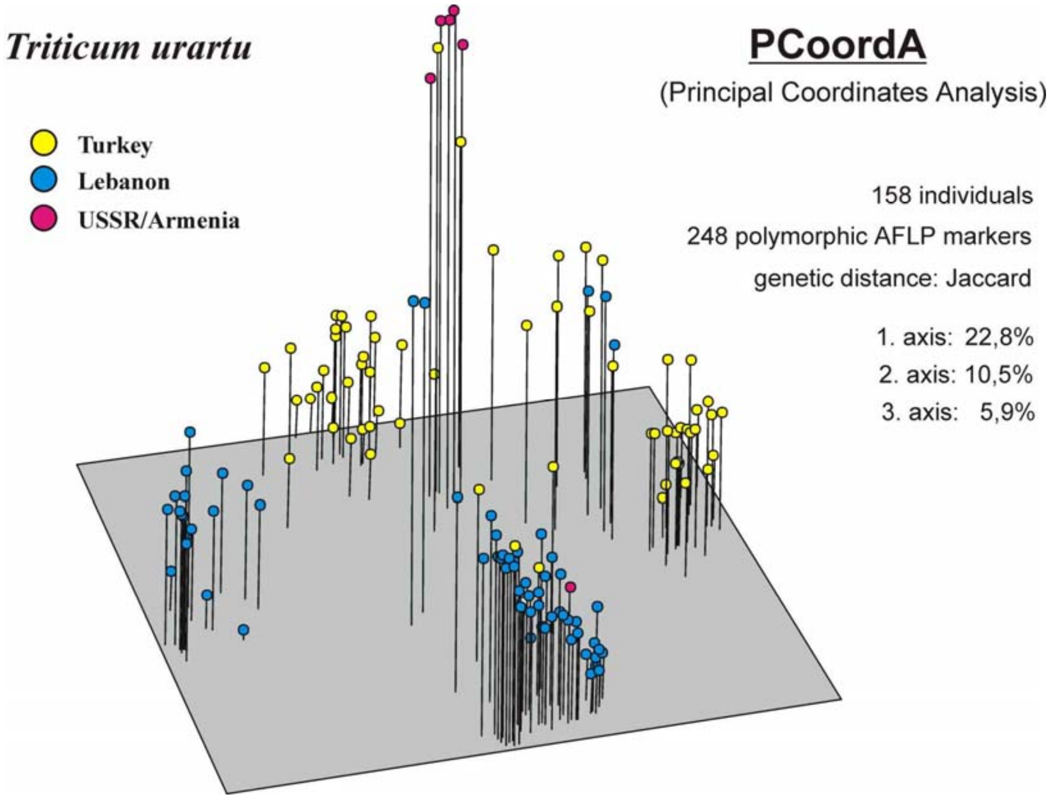

Figure S2 Principal Coordinates Analysis of the *T. urartu* wheat accessions. In this analysis, the lines molecularly intermediate between *T. urartu* and *T. monococcum* have not been considered.
