# Supplementary figures and images for: Crossability of Triticum urartu and Triticum monococcum Wheats, Homoeologous Recombination, and Description of a Panel of Interspecific Introgression Lines
Source: G3 (Bethesda). 2014 Aug 21;4(10):1931–41. doi: 10.1534/g3.114.013623 (PMC4199699; doi:10.1534/g3.114.013623)

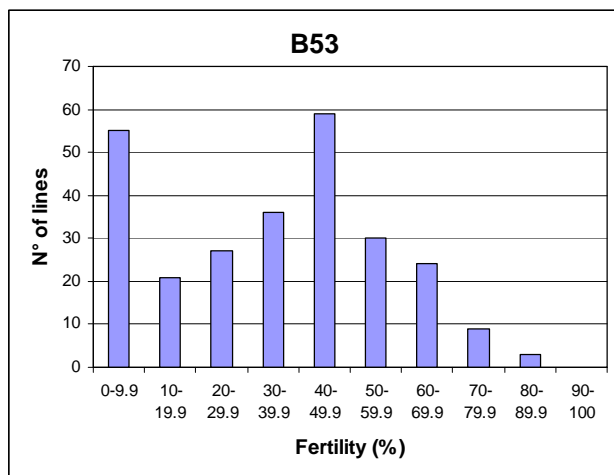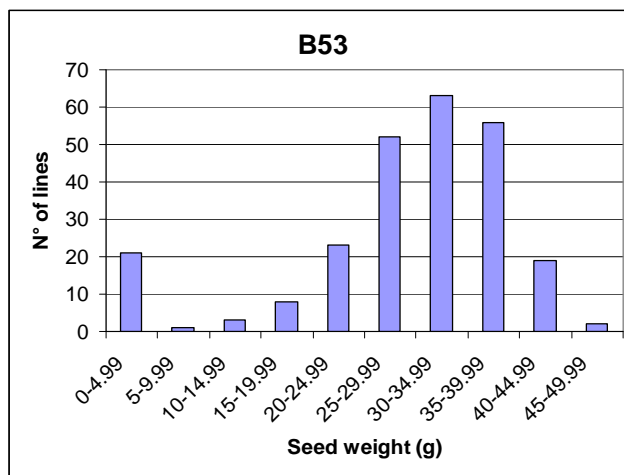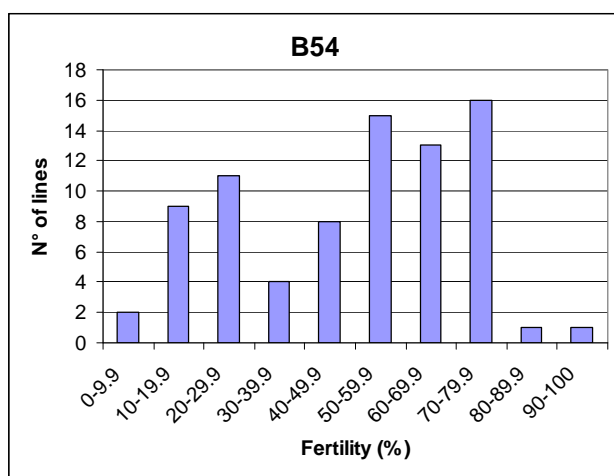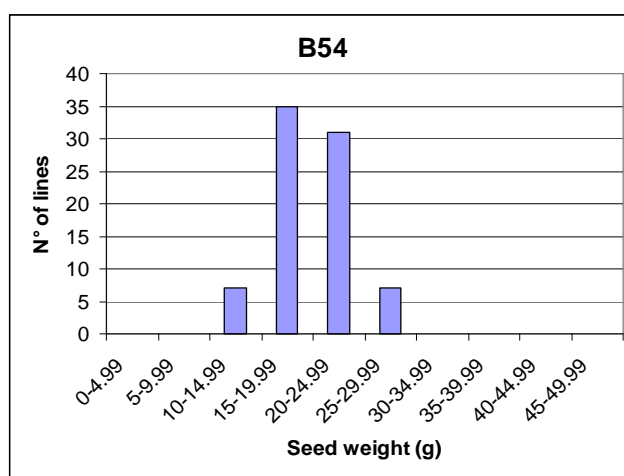

**Figure S3** Fertility (%) and 1000 seeds weight (g) distribution in the B53 (n= 268) and B54 (n= 80) populations

Supplement: Supporting Information [file supp_g3.114.013623_FigureS3.pdf]
