# Supplementary material for: Crossability of Triticum urartu and Triticum monococcum Wheats, Homoeologous Recombination, and Description of a Panel of Interspecific Introgression Lines
Source: G3 (Bethesda). 2014 Aug 21;4(10):1931–41. doi: 10.1534/g3.114.013623 (PMC4199699; doi:10.1534/g3.114.013623)
Supplement: Supporting Information [file supp_g3.114.013623_TableS2.pdf]

Table S2 Sequences of probes used for amplifying GPW microsatellites.

SSR	Sequence primer Forward (5'—3')	Sequence primer reverse (5'—3')
gpw_2006	ATGAGAAGGGGGTCAGGATT	ACATGTTCAGCCCAGGAGAC
gpw_2010	ACCCATTGCCTTTTCTTTTT	TCTGTTGATGATCCGTCCAA
gpw_2018	ATGTAGGCAGAGCACACACG	AGTCGATGAAAGGCAGCATC
gpw_2029	TAAAGCTAAACACGACGGGG	CACCGCGAACGAATTAAAAC
gpw_2032	CCTGGAAGAATAGACGTGCC	CAAGATGGGGCAGAAGATGT
gpw_2069	AGGAGAAGGCGTAAGAACCC	GGCAAGCTGGTCCTGTGTAT
gpw_2080	ATCGCATGTAACCTGCACAA	CCTTTAATCGATTGCTCGGA
gpw_2098	ACACACCCGCAAAATAATCT	TGACGCCACATAGGTCAATC
gpw_2109	TATATTGTCCACGGGGCTTC	TGGTGGAGAGTCTTGCACTG
gpw_2111	AAATTTTTGTCTGCCGGCTT	CTTGTGCGTTGAGAGTTGGA
gpw_2115	TTACAAGGCCGTAAATTGCC	TGCTTGCTGACCACTGAATC
gpw_2117	TGGCCTGAAATCTTAGCCTG	CAAGAATGCGATAAGATGGGA
gpw_2125	GGATGGGAAATGTTTGGATG	AAAATCAAACGGCAACTTGG
gpw_2127	GACAACACCGATCCGTCAC	TGTCCATGCGTTCTATTCCA
gpw_2132	TCCCGCAAATATGTGGCTAT	TATGTTGCATTGTGGTGGCT
gpw_2138	ATAGGAGGACTCCTGGGCTC	TTGCCTCAACTAGATCGCCT
gpw_2139	TGTTAACCCAGTTTCCTTATGC	ACACTGATGCATCCCACAAA
gpw_2140	GTCCACGTGCTAGGGAGGTA	ACATGCCCTAAGCTGCCCCA
gpw_2142	ACAACTGCTGCAGCTCCTTC	GATTAATTAAGCCAGGGACCG
gpw_2160	ATTTACGGCTCGACCACTCA	ACTGGAAAGGGGCGCAAGC
gpw_2166	GCCCCTGACATATTATTCACTGT	AAACTGGATGGTTGCATTCC
gpw_2169	GCCAGGCCATCAGTAAATTC	AATGGGCACAATTTGAGAGC
gpw_2216	ACGAGGAATTGCATCCTAGC	CAAAGTAGAAATTTATGCGCGA
gpw_2222	TCTCAGGAGCTAGCAGCACA	CTTCTGCCGATACATCCCAT
gpw_2228	TGTAGCTTCTGCATCACCAAA	CAAACTTGCGAGCTGCATTA
gpw_2229	CTGCGTGCTGCTCCTAATTT	CTCCACCGTGTCCTGGATAG
gpw_2237	CTTTGCTTGCGGTAGGAGAC	TGATCTATCAGGGTGAGCCC
gpw_2239	CAACCATATGCCCAGGAGAC	TGTTGCTGTCTGAAACAGGG
gpw_2243	GGGCAATCTGTTGGATCTGT	CCACTTCGCTGCTGATGTAA
gpw_2250	AGCCATAGATGGCCCTACCT	CACTCAATGGCAGGTCCTTT
gpw_2253	TGAGGAGAGGGGATATACGG	TTTGCGAAATCTTATTGCCC
gpw_2258	ATATAGGGCCGATGTGTGGA	GGTCAGCAAAGTCAGCCTT
gpw_2260	CATCTCTACCCGATCCCTCA	ACGCCGGTCTATTGAAAGTG
gpw_2264	TTGCTTTCCCAAATTGTGCT	GGCATTGAGAATCCAAGCAT
gpw_2266	TTTTTGCCCACACGGC	CGTGGAGGTGTCGACCTAAT
gpw_2269	CACATCAACAGGTCCTCTTCTA	CTAGCTGGTGGTGGTCTTGG
gpw_2270	GAGGTCGTTGAAGGGAAGG	ATCGGACGGCCTGAGTTATA
gpw_2275	CTGCTGAACGTTTGGAGGAT	GGCCGTCCTTTAGCTTTTGT
gpw_2276	ATAGGGTTCTTCTGTGCCCC	ACCCACAGTTGAACTTGGG
gpw_2277	TCAGAAGAGCGATGAGATAGAAA	GCCATTTTTAGGGCTCAGTG
gpw_2281	TCATCATGGTATGAGCGTGG	ACAAGCATTCCAATTTTGCC
gpw_2283	CTCTGTCACAACGAGCTGGA	AATGGGCTTCAGATGTCCTG
gpw_2297	TCGGAGAACCAAACTGATCC	GACTAACCACTGGGAGTCGC
gpw_2302	GCTTCACATCATAGTGTGGATAAGA	AAGCACCTCCCATGCATATC
gpw_2308	GGAGGAACCGAATCCAGAGT	GAGGCCGATCACATAAAGGA
gpw_2311	CCAAAAGTGGTGGGATCAAT	TGCAAGAACAGCTTACCGTC
gpw_2323	AGAAGTTGGCTTCCGCTTTC	AGTTGAAGATGGCCCAGATG
gpw_2328	ATCCCAACAAACACACTACCG	TTGTCTCCATGACTATGTGGG
gpw_2331	GCGGGCTCAATATTGCTAGT	GCATGGCTGAGGCTCAAGTA
gpw_2335	TTTGCAGTTGCCACAAAAGT	TGTTTTGTCTCACAGGCTGC
