# Supplementary material for: Crossability of Triticum urartu and Triticum monococcum Wheats, Homoeologous Recombination, and Description of a Panel of Interspecific Introgression Lines
Source: G3 (Bethesda). 2014 Aug 21;4(10):1931–41. doi: 10.1534/g3.114.013623 (PMC4199699; doi:10.1534/g3.114.013623)
Supplement: Supporting Information [file supp_g3.114.013623_TableS3.pdf]

Table S3 List of the introgression lines created in this study. For each introgression line the left and right markers delimiting the chromosome segments of *T. urartu* are reported. The intervals where each chromosome segment is anchored in the linkage map of *T. monococcum* are also reported.

Linkage Group	Left Marker	Right Marker	Interval (cM)	Zygosity	IL Name
4	Xgpw2279	Xcfa2173	3.59	Hetero	7197-16-9
4	Xcfa2256	Xgpw2138	4.08	Hetero	7197-16-8
3	Xwmc150a	Xbarc67	21.4	Hetero	7197-16-7
3	Xgpw2132	Xbarc218	6.5	Hetero	7197-16-6
2	Xgwm515	Xgwm1045	3.62	Hetero	7197-16-4
2	Xgpw1162	Xgpw2089	4.0	Homo	7197-16-3
1	Xgpw2005	Xcfa2226	5.8	Hetero	7197-16-2
7	Xwmc405	Xcfa2174	18.94	Hetero	7197-16-12
7	Xcfd31	Xcfa2049	16.47	Hetero	7197-16-11
7	Xcfd31	Xcfa2049	16.47	Hetero	7197-16-10
1	Xgpw2277	Xcfa2158	10.93	Hetero	7197-16-1
7	Xcfd31	Xwmc479	5.19	Homo	7189-8-8
1	Xcfd58	Xgpw2277	22.13	Homo	7189-2-2
5	Xcfa2086	Xwmc74	14.46	Homo	7189-10-6
1	Xgwm1104	Xgpw2277	5.38	Homo	7189-10-4
5	Xgwm443	Xgwm154	15.63	Homo	7189-10-3
5	Xbarc124b	Xcfa2141	5.32	Homo	7189-10-3
7	Xcfd6	Xcfa2174	4.96	Homo	7189-10-14
5	Xgwm126	Xwmc74	18.27	Homo	7189-10-13
1	Xcfd58	Xgpw2181	9.07	Homo	7189-10-12
1	Xbarc9	Xbarc9	16.12	Homo	7189-10-12
5	Xgpw2098	Xcfa2163	155.33	Homo	7189-10-12
5	Xcfa2141	Xwmc74	61.71	Homo	7188-1-2
3	Xcfa2134b	Xcfa2134a	84.9	Homo	7188-1-1
5	Xcfd39	Xwmc74	25.53	Homo	7183-8-2
1	Xcfd58	Xgpw2181	9.07	Homo	7183-5-1
1	Xcfa2158	Xgpw2005	1.1	Homo	7183-5-1
5	Xcfd2b	Xgwm271	35.61	Homo	7183-5-1
6	Xcfd190	Xwmc96a	2.88	Homo	7183-3-1
3	Xwmc147	Xcfd79	53.9	Homo	7183-2-2
4	Xwmc89	Xcfa2173	31.11	Homo	7183-2-2
3	Xcfa2134b	Xcfa2134b	14.7	Homo	7183-2-1
3	Xcfa2134b	Xcfa2134b	14.7	Homo	7183-1-2
3	Xwmc147	Xwmc147	15.5	Homo	7183-1-1
3	Xcfa2134b	Xcfa2134b	14.7	Homo	7181-1-2
7	Xwmc405	Xwmc405	2.0	Homo	7180-3-4
5	Xgwm271	Xcfa2141	28.91	Homo	7179-3-3
5	Xbarc124b	Xcfa2141	5.32	Homo	7179-3-2
5	Xcfd39	Xgwm126	7.26	Homo	7179-1-4
1	Xgwm33	Xcfd58	1.74	Homo	7178-6-1
2	Xgwm726	Xwmc177	20.74	Homo	7178-6-1
2	Xgwm726	Xwmc177	20.74	Homo	7178-4-1
2	Xgpw2125	Xbarc124a	5.48	Homo	7178-3-1
	Xwmc264	Xgwm372	21.98	Homo	7178-3-1
1	Xcfd58	Xgpw2181	9.07	Homo	7178-1-1

7	Xcfd6	Xcfa2174	4.96	Homo	7178-1-1
2	Xgpw2127	Xwmc177	43.94	Homo	7177-9-1
3	Xcfa2134b	Xgwm493	21.7	Homo	7177-16-6
2	Xgwm515	Xgwm1045	3.62	Hetero	7177-16-5
3	Xcfd79	Xwmc527	78.0	Homo	7177-16-5
2	Xgpw2125	Xbarc124a	5.48	Homo	7177-16-4
2	Xgwm275	Xwmc474	2.56	Homo	7177-16-4
2	Xgpw2281	Xgwm30	5.09	Homo	7177-16-3
3	Xcfd79	Xwmc527	78.0	Homo	7177-16-3
2	Xgpw2125	Xbarc124a	5.48	Homo	7177-16-1
2	Xgwm275	Xwmc474	2.56	Homo	7177-16-1
2	Xgpw2281	Xgwm30	5.09	Homo	7177-16-1
3	Xcfa2134b	Xgwm493	21.7	Homo	7177-16-1
3	Xcfa2134a	Xgwm1121	3.1	Homo	7177-16-1
2	Xwmc474	Xgwm515	3.82	Homo	7177-15-3
2	Xgpw2281	Xgwm30	5.09	Homo	7177-15-3
7	Xwmc405	Xwmc405	2.0	Homo	7176-11-1
4	Xwmc89	Xcfd71	3.26	Homo	7138-5-2
