# Supplementary material for: Crossability of Triticum urartu and Triticum monococcum Wheats, Homoeologous Recombination, and Description of a Panel of Interspecific Introgression Lines
Source: G3 (Bethesda). 2014 Aug 21;4(10):1931–41. doi: 10.1534/g3.114.013623 (PMC4199699; doi:10.1534/g3.114.013623)
Supplement: Supporting Information [file supp_g3.114.013623_TableS4.pdf]

Table S4 Supplementary information for Figure 2

B53

1a: 7 bands homozygous mono (m404013, m355907, m366206, m376005, m326003, m364018, m376103)
1b: 6 bands from urartu in 31.1cM (u413346, u413836, u4038e, u413848, u404028, u373829*)*
1c: 3 bands from mono in 28.6 cM (m403255, m356001, m413851)
1d: 6 bands homozygous mono (m356006, m356104, m363625, m403212d, m4133b, m423301)
1e: 1 band from mono, 1 band from urartu in 6.4 cM (u364028, m423330); m and u bands in COUPLING
1f: 4 bands from mono in 26.8 cM + 4 bands from urartu in 29.2 cM (m374026, m403251, u413343, m423229, m403215f, u403846, u424040, u403250); m and u bands in REPULSION
2a: 10 bands homozygous mono (m363818, m374015, m356108, m413812b, m374019, m364009, m414014, m373807, m376109, m423321)
3a: 1band homozygous mono (m403224)
3b: cluster of 3 bands from mono in 10.8 cM (m404025, m373820, m424041)
3c: cluster of 9 bands from urartu in 18.9 and 5 bands from mono in 11.8 cM (u413340, u404032, u403851, u413842, u414030, u373821, u413344, u363637, u363815, u373822, m403259, m374034, m413232, m414036, m404026)
3d: 1 band from mono and cluster of 6 bands from urartu in 1.9 cM (m423314, u373337, u363642, u363635, u363636, u423846, u424045); m and u bands in REPULSION
3e: 3 bands homozygous mono (m326101, m326112, m423319)
4a: 6 bands homozygous mono (m404017, m374806, m4240a, m373316, m374010, m403225)
4b: 3 bands in 12.3 cM, 2 from urartu, 1 from mono (u424013, u404003, m403208); m and u bands in COUPLING
4c: 7 bands from mono in 18.7 cM (m423234, m413838, m373824, m373332, m374024, m413234, m413204)
5a: 1 band homozygous mono (m403835)
5b: 3 bands from mono in 12.5 cM + 2 bands from urartu in 4.6 cM (m364029, m424034, m404029, u404028, u373829*)*
5c: 7 bands homozygous mono (m356113, m413815, m355902, m413328, m363819, m373211, m373304)
5d: cluster of 12 bands from urartu in 10.4 cM + 4 bands from mono in 15.5 cM (u413837, u373342, u374032, u413342, u424037, u423844, u374027, u374028, u374031, u403262, u403843, u363633, m364030, m356109, m364024, m363632)
5e: 3 bands from mono in 8.9 cM + 3 bands from urartu in 12.4 cM (m326008, m413833, m413846, u413839, u363643, u374029)
6a: 5 bands homozygous mono (m363805, m373208, m363812, m3733b, m403217)
6b: 12 bands from mono in 63.2 cM + 4 bands from urartu in 20.7 cM (m424003, m373334, m366212, m364027, m364031, m326105, m374035, m423328, m404020, m403254, m413224, m414037, u423802, u424046, u374036, u414031)
6c: 2 bands from mono in 21 cM (m326111, m413306)
6d: 5 bands homozygous mono (m374808, m403818, m403831, m326006, m413814d)
7a: 2 bands from mono in 8.2 cM (m423806, m403247)
7b: 5 bands in from urartu in 37.9cM (u403841, u373828, u424039u413235, u364035)

B54

1a: 1 band from urartu and 2 bands from mono in 24 cM (m413819, m403836, m424030) IN REPULSION
1b: 3 bands from mono in 22 cM (m364018, m363625, m4133b)
1c: 1 band from urartu (m363629)
2a: 1 band homozygous from mono (m374015)
2b: 2 bands from urartu and 6 bands from mono in 50 cM (m374014, m364009, m403809, u3738b, u364001, m373807, m374003, m403821) IN REPULSION
2c: 1 band homozygous from urartu (m413818)
3a: 2 bands from urartu in 29 cM (m364001, m364004)
3b: 1 band from mono (m3738a)
3c: 2 bands from urartu in 9 cM (u413310, m4133a)
3d: 5 bands from urartu in 43 cM (u413304, u414801, u413307, u373301, u374008)
3e: 1 band from urartu (u413314)
4a: 8 bands from mono in 35 cM (m404017, u364004, m4240a, m374010, u364007, m373316, m364012, m413334)
4b: 1 band from mono (m374024)
5a: 2 bands homozygous from mono in 38 cM (m403835, m424034)
5b: 1 band from mono and 1 band from uraru in 17 cM (m4138c, m4038b) IN REPULSION
5c: 1 band from mono (m373304)
5d: 11 bands from urartu in 48 cM (u373806, u403808, u373809, u413801, u413805, u373302, u374004, u413303, m404006, u373813, u373802)
5e: 1 band from mono (m413326)
5f: 1 band homozygous from urartu (m413825)
6a: 1 band from mono (m3738b)
6b: 7 bands from mono in 44 cM (m4038c, u41339, u40389, m413820, m4038a, u363605, m413306)
6c: 4 bands from mono in 52 cM (u40387, m414806, u373810, u364008)
7a: 1 band homozygous from mono (m414810)
7b: 3 bands from mono in 19 cM (m3740a, m423806, u37383)

* Assigned to both 1b and 5b groups of B53 with a LOD > 3.0; preferentially assigned to group 1b with a LOD > 4.0
