# Supplementary material for: Crossability of Triticum urartu and Triticum monococcum Wheats, Homoeologous Recombination, and Description of a Panel of Interspecific Introgression Lines
Source: G3 (Bethesda). 2014 Aug 21;4(10):1931–41. doi: 10.1534/g3.114.013623 (PMC4199699; doi:10.1534/g3.114.013623)
Supplement: Supporting Information [file supp_g3.114.013623_FigureS1.pdf]

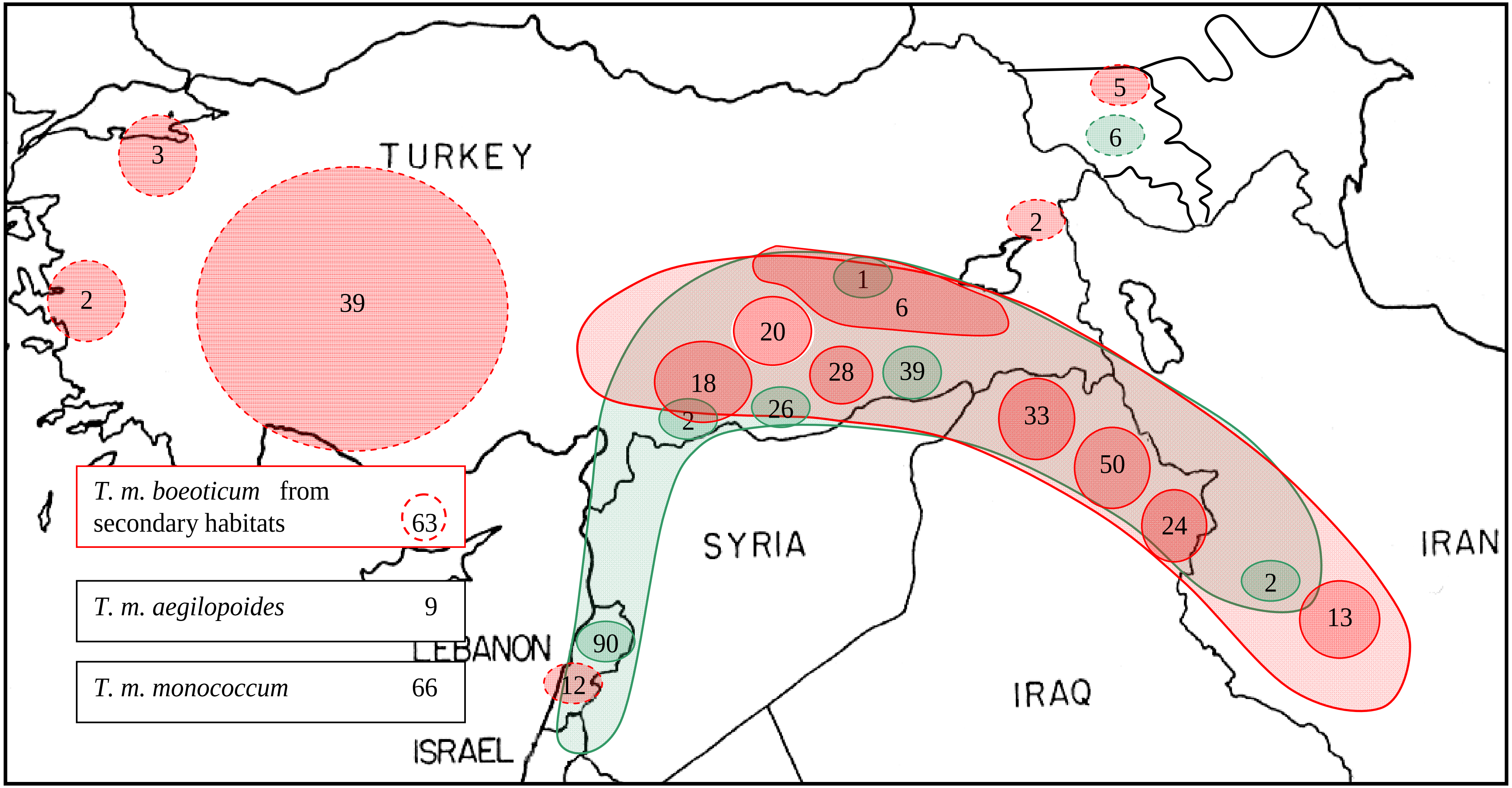

Figure S1 Geographic distribution and number of AFLP-fingerprinted *Triticum monococcum* ssp. *boeoticum* (red circles) and *Triticum urartu* (green circles) accessions. The red- and the green-delimited areas correspond to the primary habitats of *T. m.* subsp. *boeoticum* (Heun et al., 1997) and of *T. urartu* (see Valkoun et al., 1998), respectively. The number of *T. m.* ssp. *boeoticum* from secondary habitats, as well as ssp.*monococcum* and ssp. *aegilopoides* acccessions, is also reported.
